# Artificial intelligence for topic modelling in Hindu philosophy: Mapping themes between the Upanishads and the Bhagavad Gita

**DOI:** 10.1371/journal.pone.0273476

**Published:** 2022-09-01

**Authors:** Rohitash Chandra, Mukul Ranjan

**Affiliations:** 1 Transitional Artificial Intelligence Research Group, School of Mathematics and Statistics, UNSW, Sydney, Australia; 2 Department of Electronics & Electrical Engineering, Indian Institute of Technology Guwahati, Guwahati, Assam, India; Hong Kong Metropolitan University, HONG KONG

## Abstract

The Upanishads are known as one of the oldest philosophical texts in the world that form the foundation of Hindu philosophy. The Bhagavad Gita is the core text of Hindu philosophy and is known as a text that summarises the key philosophies of the Upanishads with a major focus on the philosophy of karma. These texts have been translated into many languages and there exist studies about themes and topics that are prominent; however, there is not much done using language models which are powered by deep learning. In this paper, we use advanced language models such as BERT to provide topic modelling of the Upanishads and the Bhagavad Gita. We then map those topics of the Bhagavad Gita and the Upanishads since it is well known that Bhagavad Gita summarizes the key messages in the Upanishads. We also analyse the distinct and overlapping topics amongst the texts and visualise the link of selected texts of the Upanishads with the Bhagavad Gita. Our results show very high similarity between the topics of these two texts with the mean cosine similarity of 73%. We find that out of the fourteen topics extracted from the Bhagavad Gita, nine of them have a cosine similarity of more than 70% with the topics of the Upanishads. We also find that topics generated by the BERT-based models show very high coherence when compared to the conventional models. Our best-performing model gives a coherence score of 73% on the Bhagavad Gita and 69% on the Upanishads. The visualization of the low-dimensional embeddings of these texts shows very clear overlapping themes among their topics adding another level of validation to our results.

## 1 Introduction

*Philosophy of religion* [1–4] is a field of study that covers key themes and ideas in religion and culture that relate to philosophical topics such as ethics and metaphysics. Hindu philosophy [5–7] consists of schools developed for thousands of years which focus on themes such as ethics [8], consciousness [6], karma [9, 10], logic and ultimate reality (Brahman) [7]. Hindu philosophy is at times referred as Indian philosophy [11, 12]. The philosophy of karma and reincarnation is central to Hindu philosophy [12]. The Upanishads form the key texts of Hindu philosophy and seen as the conclusion of the Vedas [13–17]. Hindu philosophy [11] consists of six major theistic (Astika) schools include Vedanta [16], Samkhya [18], Nyāya [19], Vaisheshika [20], Mīmāmsā [21], and Yoga [22]. Moreover, Jain [23], Buddhist [24, 25], Carvaka [26] and Ājīvika [27] philosophy are the major agnostic and atheistic (Nastika) schools of Hindu philosophy. There has been a lot of interest in Hindu philosophy, particularly in the west, with a large list bibliography of translations of key texts such as the Upanishads [28]. Moreover, Hindu and Buddhist philosophy have parallels with development of specific themes in Greek philosophy [29].

The Upanishads and Bhagavad Gita are the foundational texts for Hindu philosophy. A distinct feature of Hindu religious and philosophical texts is that they come from a library of texts rather than a single source. These texts have been written much later in verse form in Sanskrit language, they have been sung and remembered for thousands of years in the absence of a writing system [13]. The Bhagavad Gita is part of the Mahabharata which is known as the one of oldest and largest epics written in verse in the Sanskrit language [30–32]. The Bhagavad Gita is known as a concise summary of Hindu philosophy [12] with a major attribute which is the philosophy of karma [33–35]. The Upanishads is a collection of philosophical texts of ancient India which marks the foundation in the history of philosophy [36]. There are 108 books of the Upanishads of which most were lost in time, and then re-written. There are 12 prominent books of the Upanishads which have been well studied by Hindu and western scholars [28, 37].

Nowadays, deep learning is the backbone of natural language processing (NLP) methods [38–40]. NLP considers tasks such as topic modelling, language translation, speech recognition, semantic and sentiment analysis [39]. Sentiment analysis provides an understanding of human emotions and affective states [41–43]. Recurrent neural networks such as long-short term memory (LSTM) network models have been prominently used as language models due to their capability to model temporal sequences [44]. LSTM models have been improved for language modelling using attention-based mechanisms [45], and encoder-decoder LSTM framework with attention (Transformer) [46, 47]. Bidirectional encoder representations from Transformer (BERT) [48] model is a pre-trained language model that features more than 300 million model parameters for language modelling tasks. Topic models help us better understand a text corpus by extracting the hidden topics. Traditional topic models such as linear discriminant analysis (LDA) [49] assume that documents are a mixture of topics and each topic is a mixture of words with a certain probability score. Sentence BERT (S-BERT) [50] improves BERT model by reducing computational time to derive semantically meaningful sentence embedding. Recent topic modelling frameworks use S-BERT for embedding in combination with clustering methods [51–56]. BERT-based models have shown promising results for topic modelling [52, 56–58], which motivates their usage in our study.

Religious linguistics refer to the study of religious sentences and utterances [59]. The major aim of the religious linguistic research is to create an analysis of various subject matters related to religious sentences which include God, miracles, redemption, grace, holiness, sinfulness along with several other philosophical interpretations [60–62]. Most translations of the Bhagavad Gita and related texts come with interpretations and commentary regarding philosophy and how the verses relate to issues at present [63]. Stein [64] presented a study about multi-worded expressions by extracting local grammars based on semantic classes in the Spanish translation of the Bhagavad Gita and found it to be promising for understanding religious texts and their literary complexity. The role of multi-word expressions (MWE) could be a way to better understand the metaphorical and lyrical style of the Bhagavad Gita. Rajendran [65] presented a study on metaphors in Bhagavad Gita using text analysis based on conceptual metaphor theory (CMT). The analysis identified the source and target domains for the metaphors, and traced the choice of metaphors to physical and cultural experiences. The metaphors have been inspired by the human body and ancient India, which resonate with modern times. Rajput et al. [66] provided a statistical study of the word frequency and length distributions prevalent in the translations of Bhagavad Gita in Hindi, English and French from the original composition in Sanskrit. The Shannon entropy-based measure estimated the vocabulary richness with Sanskrit as the highest, and word-length distributions also indicated Sanskrit having the longest word length. Hence, the results demonstrated the inflectional nature of Sanskrit. Dewi [67] studied metaphorical expressions and the conceptual expression underlying them by reviewing 690 sentences related to metaphor of life from Bhagavad Gita and analyzed them using some conceptual metaphor theory. It was reported that the Bhagavad Gita featured 24 conceptual metaphors among which *life is an entity*, *life is a journey* and *life is a continuous activity* are the most frequent ones. Bhuwak [68] examined specific ideas from Bhagavad Gita such as cognition, emotion, and behaviour by connecting them with the context of human desire. It was reported that desires lead to behaviour and achievement or non-achievement of desire leads to positive and negative emotions which can be managed in a healthy way by self-reflection, contemplation and the practice of *karmayoga* (selfless action). In our earlier work, the BERT-based language model framework was used for the sentiment and semantic analysis as a means to compare three different Bhagavad Gita translations. We found that although the style and vocabulary differ vastly, the semantic and sentiment analysis shows similarity in the meaning of the majority of the verses [69]

Although the Bhagavad Gita and Upanishads have been translated into a number of languages and studies about their central themes and topics have been prominent, there is not much work in utilising the latest advancements from artificial intelligence, such as topic modelling using language models—powered by deep learning. In this paper, we use advanced language models such as BERT in a framework to provide topic modelling of the key texts of the Upanishads and the Bhagavad Gita. We analyse the distinct and overlapping topics amongst the texts and visualise the link of selected texts of the Upanishads with the Bhagavad Gita. Our major goal is to map the topics in the Bhagavad Gita with the Upanishads; since it is well known that the Bhagavad Gita summarizes the key messages in the Upanishads, and there are studies about the parallel themes in both texts [70]. We also provide a comparison of the proposed framework with LDA which has been prominent for topic modelling.

The rest of the paper is organised as follows. In Section 2, we give a background about the Bhagavad Gita and Upanishads. Section 3 presents the methodology that highlights model development for topic modelling. Section 4 presents the results and Section 5 provides a discussion and future work.

## 2 Background

### 2.1 BERT language model

BERT is an attention-based Transformer model [46] for learning contextualized language representation where the vector representation of every input token is dependent on the context of its occurrence in a sentence. The Transformer model [46] has been developed by using long short-term memory (LSTM) recurrent neural networks [44, 71] with an an encoder-decoder architecture [72]. Transformer models implement the mechanism of attention by weighting the significance of each part of the input data which has been then prominent for language modelling tasks [46, 73].

BERT is first trained to understand the language (called pre-training phase) and the context after that it is fine-tuned to learn the specific task such as neural machine translation (NMT) [48, 74–78], question answering [79–84], and sentiment analysis [85–89]. The pre-training phase of BERT involve two different NLP tasks such as masked language modelling (MLM) [48, 90, 91] and next sentence prediction (NSP) [48]. MLM and NSP are semi-supervised learning tasks. In MLM, 15% words in each input sequence are randomly replaced with a *mask* token and the model is trained to predict these randomly masked input sequences based on the context provided by the neighbouring non-masked words. In NSP, the BERT model learns to predict if two sentences are adjacent to each other. In this way, a BERT model is trained simultaneously to minimize the combined loss function and hence learn the contextualized word embedding. In the fine-tuning phase, one or more fully connected layers are added on top of the final BERT layer based on the application. Since BERT is pre-trained, it can be more easily trained further with datasets for specific applications. In our earlier works, the BERT-based framework has been used for sentiment analysis of COVID-19 related tweets during the rise of novel cases in India [92]. A similar framework using BERT was used for modelling US 2020 presidential elections with sentiment analysis from tweets to predict the state-wise winners [93].

Based upon the number of transformer blocks, BERT [48] is available with two variants: 1.) *BERT*_*BASE*_ consists of 12 transformer blocks stacked on top of each other with a hidden dimension embedding of 768 and 12 Attention heads, on the other hand, 2.) *BERT*_*LARGE*_ consists of 24 transformer blocks with a hidden dimension embedding of 1024 and 16 attention heads. *BERT*_*BASE*_ has a total of 110 million parameters while *BERT*_*LARGE*_ has a total of 340 million parameters. BERT takes into account the context for each occurrence of a given word, in comparison to context-free models such as word vectors (word2vec) [94] and global vector (GloVe) [95], which generate a single word embedding representation for each word in the vocabulary.

### 2.2 Document embedding models

The *universal-sentence-encoder* [96] is a sentence embedding model that encodes sentences into high-dimensional embedding vectors that can be used for various natural language processing tasks. The model takes a variable length English text as an input and gives a 512-dimensional output vector. The model is trained with deep averaging networks (DANs) [97] encoder, which simply takes the average of the input embeddings for words and bi-grams and then passes them through one or more deep neural networks to get the sentence embeddings. *Sentence-BERT*(S-BERT) [50] extends the BERT model and Siamese and triplet network [98] to generate the sentence embeddings. S-BERT uses BERT embeddings with a pooling layer to get the sentence-embedding (*u* and *v*) of two sentences. S-BERT has been fine-tuned with objective functions such as triplet loss function and cosine similarity between *u* and *v*.

### 2.3 Clustering techniques

Clustering is a type of unsupervised machine learning that groups unlabelled data based on a given similarity measure for a given dataset *x*^(1)^, …, *x*^(*n*)^, where *x*^(*i*)^ ∈ **R**^*d*^ is a d-dimensional data point from the dataset. The goal of clustering is to assign each data point a label or a cluster identity. Although a large number of clustering algorithms exist in the literature, we select two for this study. Xu et al. [99] presented an exhaustive list of different groups of clustering algorithms that includes: 1.) centroid-based algorithms such as k-means clustering [100]; 2.) hierarchical-based algorithms such as agglomerative clustering [101] which creates a hierarchical relationship among the data points in order to cluster them; 3.) density based algorithms that connect an area with high density into clusters [102]; 4.) distribution based clustering such as Gaussian mixture model [103] that assumes that data generated from same distribution belongs to the same clusters.

*K-means clustering* [104] clusters n-data points into k-clusters, where each data point belongs to the cluster with the nearest mean. The k-means algorithm can be explained in three steps. The first step involves the initialization of the k-centroid corresponding to each cluster. In the second step, a point is assigned to the closest cluster centroid. In the third step, the centroid for each cluster is recalculated based on new assigned data points and step 2 and 3 is repeated till convergence.

*Hierarchical density-based spatial clustering of application with noise (HDBSCAN*) [105, 106] is a density-based hierarchical clustering algorithm that defines clusters as highly dense regions separated by sparse regions. The goal of the algorithm is to find high probability density regions which are our clusters. It starts with estimating the probability density of the data by using the distance of the *k*^*th*^ nearest neighbours, defined as the core distance *core*_*k*_(*x*). If a region is dense, then the distance of *k*^*th*^ nearest neighbour will be less since more data points will fit in the region of a small radius. Similarly, for the sparse region, a larger radius would be used. We define a distance metric called *mutual-reachability-distance* between two points *a* and *b* in order to formalize the concept of density (Eq 1).

$$\begin{array}{c} d_{\text{mreach-}k}\left(a,b\right)=max\left\{{\text{core}}_{k}\left(a\right),{\text{core}}_{k}\left(b\right),d\left(a,b\right)\right\} \end{array}$$ (1)

where, *d*(*a*, *b*) gives the euclidean distance between point *a* and *b*. This mutual reachability distance is used to find the dense areas of the data but since the dense areas are relative and different clusters (dense areas) can have different densities. The entire data points can be modelled as a weighted graph with weight *d*_*mreach*−*k*_(*a*, *b*) of the edge between nodes *a* and *b*.

### 2.4 Dimentionality reduction techniques

*Uniform manifold approximation and projection(UMAP)* [107] for dimension reduction is a non-linear dimensionality reduction technique which is constructed from the theoretical framework based on Riemannian geometry and algebraic topology. The detailed theoretical explanation of the algorithm is out of the scope of this paper and can be seen in McInnes et el. [107]. UMAP can be used in a way similar to t-distributed stochastic neighbor embedding (t-SNE) [108] and principal component analysis (PCA) [109] for dimensionality reduction and visualization of high dimensional data.

Latent Dirichlet allocation (LDA) [49] is a generative probabilistic model for the topic modelling of the corpus based on word frequency. The basic idea behind the model is that each document is generated by a statistical generative process; hence, each document can be modelled as a random mixture of latent topics, and each topic is a mixture of words characterised by its distribution. A *word* denoted by *w* and indexed from 1 to the vocabulary size *V* and a *document* is given by **w** = {*w*_1_, *w*_2_, …, *w*_*N*_}, where *w*_*i*_ is the *i*^*th*^ word in the sequence [49]. The generative process involved in the algorithm can be summarized as 1.) fix the number of topics and hence the dimensionality of the Dirichlet distribution and that of the topic variable *z*, and sample *θ*(per-document topic proportion) from a Dirichlet prior *Dir*(*α*) 2.) sample a topic *z*_*n*_ from a multinomial distribution *p*(*θ*; *α*) and then 3.) sample a word *w*_*n*_ from multinomial probability distribution conditioned on *z*_*n*_, *p*(*w*_*n*_|*z*_*n*_, *β*). Overall probability of document **w** containing *N* words is given by Eq 2.

$$\begin{array}{c} p\left(w\right)=\int_{\theta }(\prod_{n=1}^{N}\sum_{z_{n}=1}^{k}p(w_{n}|z_{n};\beta )p(z_{n}|\theta ))p\left(\theta ;\alpha \right)d\theta \end{array}$$ (2)

Given a corpus of *M* documents *D* = {*w*_1_, …, *w*_*M*_}, the EM algorithm can be used to learn the parameters of an LDA model by maximizing a variational bound on *p*(*D*), as seen in Eq 3.

$$\begin{array}{c} \text{log}\;p\left(D\right)\geq \sum_{m=1}^{M}E_{q_{m}}\left[\text{log}\;p\left(\theta ,z,w\right)\right]-E_{q_{m}}[\text{log}\;q_{m}\left(\theta ,z\right)] \end{array}$$ (3)

LDA has been used for several language modelling tasks that include the study of the relationship between two corpora using topic modeling [110] which is also the focus of our study.

## 3 Methodology

### 3.1 Datasets

We evaluated a number of prominent translations of the Bhagavad Gita and the Upanishads. In order to maintain the originality of the themes and ideas of these two classical Indian texts, we used the older and more prominent translations for this study. We chose Eknath Easwaran’s translation since he directly translated from Sanskrit to English and translated both texts [111, 112], hence it would be not be creating a translation bias for topic modelling and comparison of the topics between the texts. Eknath Easwaran (1910–1999) was a professor of English literature in India and later moved to the United States where he translated these texts. In addition, we chose the translation by Shri Purohit Swami and William Butler Yeats [113] for further comparison. W. B Yeats (1865–1939) was an Irish poet, dramatist, and prose writer and known as one of the foremost figures of 20th-century literature. Shri Purohit Swami (1882–1941) was a Hindu teacher from Maharashtra, India. The translation of the Upanishads by them is special since it has been done jointly by prominent Indian and Irish scholars and captures Eastern and Western viewpoints. Table 1 provides further details of the texts. Note that Shri Purohit Swami also translated the Bhagavad Gita [114] which can be used in future analysis, and not used in this work.

**Table 1:** Details of the texts used for topic modelling.

Texts	Translator	Year
The Bhagavad Gita [111]	Eknath Easwaran	1985
The Upnishads [112]	Eknath Easwaran	1987
The Ten Principal Upanishads [113]	Shri Purohit Swami & W.B. Yeats	1938
108 Upanishads [115]	The International Gita Publication	–

The Bhagavad Gita consists of 18 chapters which feature a series of questions and answers between Lord Krishna and Arjuna that range with a range of topics including the philosophy of Karma. The Mahabharata war lasted for 18 days [116]; hence, the organisation of the Gita is symbolic.

*The Upanishads* [112] translated by Eknath Easwaran provides a commentary and translation of the 11 major and 4 minor Upanishads. The *108 Upanishads* [115] is a collection of the translation and commentary of all 108 Upanishads in a single book compiled by the *Gita Society*. The translation and commentary are done by a group of spiritual teachers who have tried to recover the Upanishads which have believed to be lost earlier; however, there are not much details about how they have recovered them [115]. The Chandogya Upanishad has the highest number of words followed by the Katha Upanishad and the Brihadaranyaka Upanishad. *The Ten Principal Upanishads* [113] consists of the translation of the 10 major Upanishads. This text does not have a separate explanation for each Upanishad unlike the Upanishads by Eknath Easwaran. The Brihadaranyaka Upanishad consists of the highest number of words followed by the Chandogya Upanishad and Katha Upanishads. The Chandogya Upanishad is one of the largest Upanishads consisting of 8 chapters which can be divided into 3 natural groups according to the philosophical ideas [117]. The first group (Chapter 1 and Chapter 2) deals with the structure and different aspects of the languages and their expression, particularly with the syllable “Om” that is used to describe Brahman and beyond. The second group (Chapter 3-5) consists of the ideas of the universe, life, mind and spirituality. The third group (Chapter 6-8) deals with the more metaphysical questions such as nature of reality and Self [117]. Since the first five chapters are intermixed with rituals, Shri Purohit Swami omitted them from in his translation [113] along with some passages from the Brihadaranyaka Upanishad. Other authors also state that some of the passages of the Brihadaranyaka Upanishad have been omitted due to the repetitions [113]. Brihadaranyaka Upanishad, consisting of 6 chapters discusses about different philosophical ideas including one of the earliest formulations of the Karma doctrine (Verse 4.4.5), ethical ideas such as self-restraint (Damah), charity (Danam) and compassion (Daya) and also other metaphysical topics related to the philosophy of Advaita Vedanta. Eknath Easwaran [112] translated this chapter as the *Forest of Wisdom* which starts with the one of Vedic theories of the creation of the Universe and then the dialogue between a great sage, Yajnavalkya, and his wife Maitreyi which is a deep spiritual discussion about death, possession, self, Brahman (God) and the Atman (Self). It contains one of the earliest psychological theories relating to the human body, mind, ego and the Self. The Katha Upanishad is one of the legendary stories of a small boy Nachiketa who met Yama (the god of Death) and asks him different questions about the nature of life, death, man, knowledge, Atman and Moksha (liberation). The Katha Upanishad consists of 2 chapters each consisting of 3 sections.

### 3.2 Framework

Our major goal is to map the topics in the Bhagavad Gita with Upanishads. We begin by selecting 12 prominent Upanishads *(Isha, Katha, Kena, Prashna, Munda, Mandukya, Taittiri, Aitareya, Chandogya, Brihadaranyaka, Brahma, Svetasvatara)* from the text translated by Eknath Easwaran [112]. The major reason that we selected both by the same author for this task is to eliminate any bias in translation for topic modelling. However, we also considered other translations as mentioned in Table 1 and found that the bias does affect the similarity matrix. For example, when we compared the similarity between the Upanishads and the Bhagavad Gita by Eknath Easwaran (same translator in both texts), the average similarity score was 3% better than that of the Bhagavad Gita by Eknath Easwaran and the Upanishads by Shri Purohit Swami (different translators in both texts). Finally, we also present the visualization of the topic space of 108 Upanishads based on the Vedas from where the Upanishads originated. Note that the Upanishads are also known as the concluding chapters of the Vedas.

**Fig 1:**
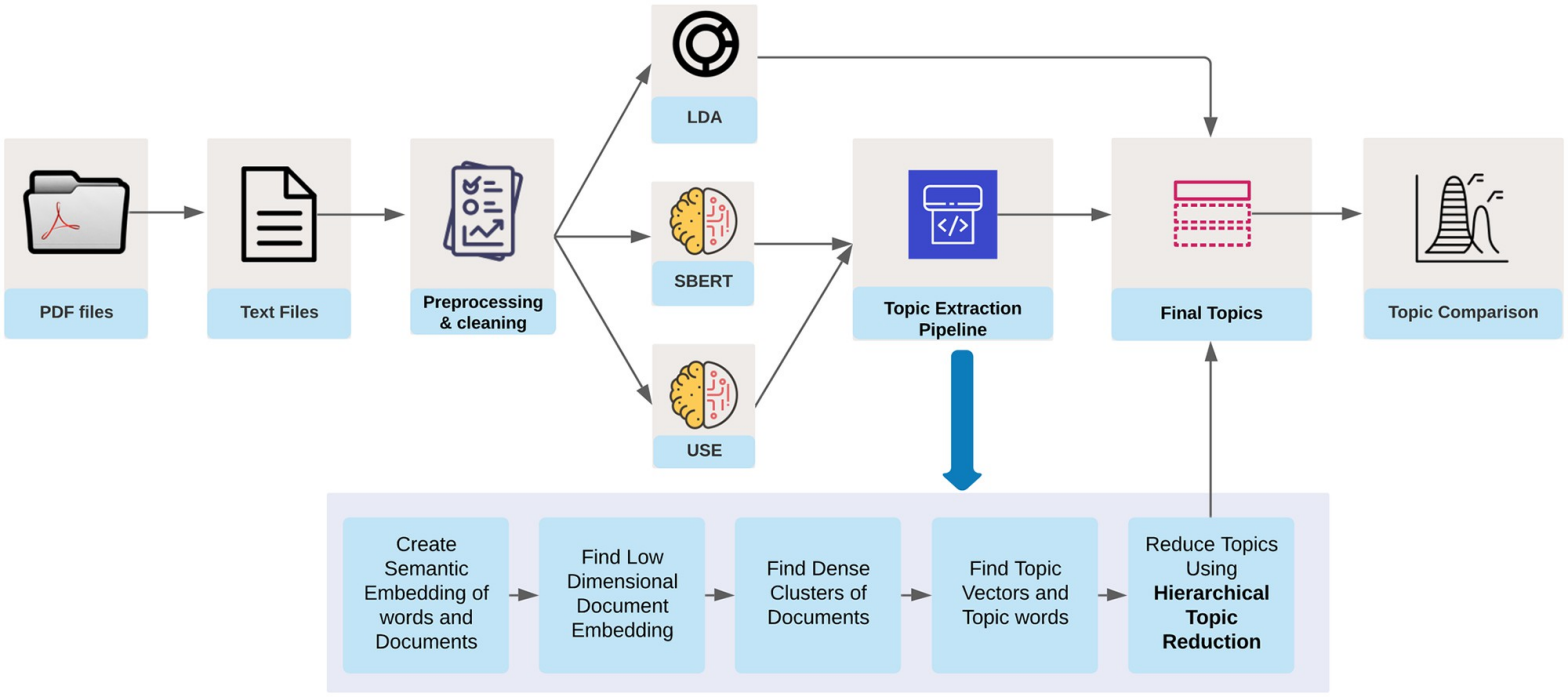
Topic modelling framework for comparison of the Upanishads with the Bhagavad Gita.

Next, we present a framework that employs different machine learning methods for topic modelling. Fig 1 presents the complete framework for the analysis and topic modelling of the respective texts given in Table 1. In Fig 1, the first stage consists of the conversion of PDF files and text pre-processing as discussed in the previous section. In the second stage, we use two different sentence embedding models 1.) universal sentence encoder (USE) and 2.) Sentence-BERT(S-BERT) for generating the word and documents embedding which is later passed through the topic extraction pipeline to generate the topic vector and finally, we compare our results with the classical topic modelling algorithm LDA [49] across the different corpora. Our framework to generate topics is similar to Top2Vec [54]; however, we also used other clustering algorithms. First, we use S-BERT and USE to generate the joint semantic embedding of documents and words. These embeddings are generally in a higher dimension which is very sparse; hence, we need to reduce the dimension of the embedding to get the dense areas. We use dimensionality reduction techniques such as UMAP and PCA for reducing the high dimensional embedding vectors generated by the S-BERT and the USE. We then find dense clusters of topics in the document vectors of the corpus using algorithms such as HDBSCAN and k-means clustering. The clusters are represented by the centroid of document vectors in the original dimension, which is called topic vectors [54]. Finally, we find the top N (N = 50 in our case) nearest words for the topic vectors that represent our final topic. Topic vectors also allow us to group similar topics and hence reduce the number of topics using *hierarchical topic reduction* [54].

Most of the topic modelling research [54, 118, 119] involves the bench-marking model results on pre-existing datasets such as the *20 News Groups dataset* [120], the *Yahoo Answers dataset* [121, 122], *Web Snippets dataset* [123], *W2E datasets* [124]. These datasets have been prepared to be used for the algorithm bench-marking tasks and consist of a fixed number of documents and words. The *20 News Groups Datasets*, for example, consists of 15,465 documents and 4,159 words [118]. Tweets have also been used for topic modelling tasks [125–127]. Jonsson et al. [125] for example, collected tweets from Twitter to prepare a datasets of 129,530 tweets and used LDA [49], *Biterm-Topic-Model*(BTM) [126] and a variation of LDA algorithms for topic modelling to compare their performance. In the case of Twitter-based topic modelling datasets, a tweet is considered as a *Document* which can be a problem for topic modelling. Jonsson et al. [125] aggregated documents to form *pseudo-documents* and found that it solves the poor performance of LDA on shorter documents. Murakami et al. [128] used research papers published in the journal *Global Environmental Change (GEC)* from the first volume (1990/1991) to Volume 20 (2010) as the corpus for the topic modelling. They divided a paper into several paragraph blocks and modelled them as documents of the corpus.

The Bhagavad Gita and Upanishads are written in verse form and to maintain the originality of the texts, most of the translations also preserve the numbering of the verses. Other than the verses, the translations also contain commentary by the translator of the texts. While creating the datasets, we first created documents based on the verse number in the texts, i.e a verse is considered as a *document of the corpus*, where the numbering is clearly mentioned. In other cases when verse numbers are not mentioned clearly, we considered one paragraph as one document. In the case of the commentary, we split the commentary into smaller parts to make them a *document* as done by Murakami et al. [128]. The statistics in terms of the number of documents, the number of words (# words), the average number of words (avg # words), and the number of verses (# verses) of the different corpus (text files) and their details can be found in Table 3.

### 3.3 Text data extraction and processing

In order to process the files given in printable document format (PDF), we converted them into text files. Most of the PDF files were generated from the scanned images of the printed texts, hence we used optical character recognition (OCR) based open-source library ocr2text. This conversion from PDF to text file gave us a raw dataset consisting of all the texts shown in Table 1. Next, pre-processing is done on the entire datasets, which consists of the following steps.

1. Removing Unicode characters generated in the text files due to noise in the PDF files;
2. Normalizing(assigning uniform verses from each text) verse numbering in the Upanishads and the Bhagavad Gita;
3. Replacing the archaic English words such as “thy” and “thou” with modern English words like your and you;
4. Removing the punctuation, extra spaces, and lower-casing;
5. Removing repetitive and redundant sentences such as “End of the Commentary”.

Examples of selected text from the original document along with the processed text are shown in Table 2. In topic modelling literature, *word* is the basic unit of data which is defined to be an item from vocabulary indexed by {1, …, *V*}, where *V* is the vocabulary size. A *Document* is a collection of *N* words represented by **w** = {*w*_1_, *w*_2_, …, *w*_*N*_}, where *w*_*i*_ is the *i*^*th*^ word in the sequence. The corpus is considered as a collection of *M* documents denoted by *D* = {**w**_1_, **w**_2_, …, **w**_*M*_} [49].

**Table 2:** Processed text after removing special characters and transforming archaic words into modern English.

Original Documents	Transformed Documents
II-5(a). What winds up empirical life is (its) appearance as unreal.	what winds up empirical life is its appearance as unreal.
“What discipline is required to know, \u2018this is a pot, except the adequacy of the means of right \u2019 \n knowledge?”	the adequacy of the means of right knowledge.”
Lord, have we not prophesied in thy name? and in thy name have cast out \n devils? and in thy name done many wonderful works?	Lord have we not prophesied in your name and in your name have cast out devils and in your name done many wonderful works.

### 3.4 Technical details

In our framework, S-BERT and USE are used for the task of generating sentence embedding. We used pre-trained S-BERT, which has been trained on a large multilingual corpus. The model uses distilled BERT (DistilBERT) [129] which is a light Transformer model trained by distilling BERT base. The output is pooled using an average pooling layer, and a fully connected (dense) layer is used finally to give a 512-dimensional output. We use different combinations of dimensionality reduction techniques and clustering algorithms with the pre-trained semantic embedding to get the final topics for each corpus.

The embedding dimension is reduced to the 5 dimensions using the selected dimensionality reduction techniques i.e UMAP and PCA. UMAP uses two important parameters, *n_neighbors* and *min_dist* in order to control the local and global structure of the final projection. We fine-tuned these parameters to optimize the topic-coherence metric and use the final UMAP model with the default *min_dist* value of 0.1, *n_neighbors* value of 10 and the *n_components* value of 5, which is the final dimension of the embeddings. We set the *random-state* to 42 and use *cosine-similarity* as the distance metric.

After getting the embedding of the documents in the reduced dimensions, we use two different clustering algorithms (HDBSCAN and k-means), where each cluster represents a topic. We fine-tuned different parameters of HDBSCAN to get the optimal value of the topic coherence metric which represents the quality of the topics found. We choose the number of topics obtained at the optimal value of the topic coherence metric as the optimal number of topics and used the same number as the value of *k* for k-means clustering. The *min_cluster_size* defines the smallest grouping size to be considered as cluster and we set it to 10. Finally, in the remaining two parameters, we use *metric* = *euclidean* and *min*_*samples* = 5. We train the k-means algorithm for 300 iterations (default in the library), with the same value for *k* as the number of labels found using HDBSCAN.

## 4 Results

### 4.1 Data analysis

We begin by reporting key features of the selected texts (datasets) as shown in Table 3. *The Upanishads* by Eknath Easwaran contains 862 documents, 40737 words and 705 verses. The text contains accompanying explanation/interpretation text by the author as well; hence, the number of documents is more than the number of verses. The *Ten Principal Upanishads* by W. B. Yeats and Shri Purohit Swami consists of 1267 documents. The corpus consists of 27492 words with an average of 21.70 words per document. The *Bhagavad Gita* by Eknath Easwaran consists of 700 verses and the same number of documents along with 20299 words with an average of 21.70 words per document.

**Table 3:** Dataset statistics.

Corpus	# Documents	# Words	Avg # words	# Verses
The Upanishads(Eknath Easwaran)	862	40737	47.26	708
The Bhagavad Gita(Eknath Easwaran)	700	20299	27.50	700
Ten principal Upanishads	1267	27492	21.70	1267
108 Upanishads	6191	405559	65.50	6191

**Fig 2:**
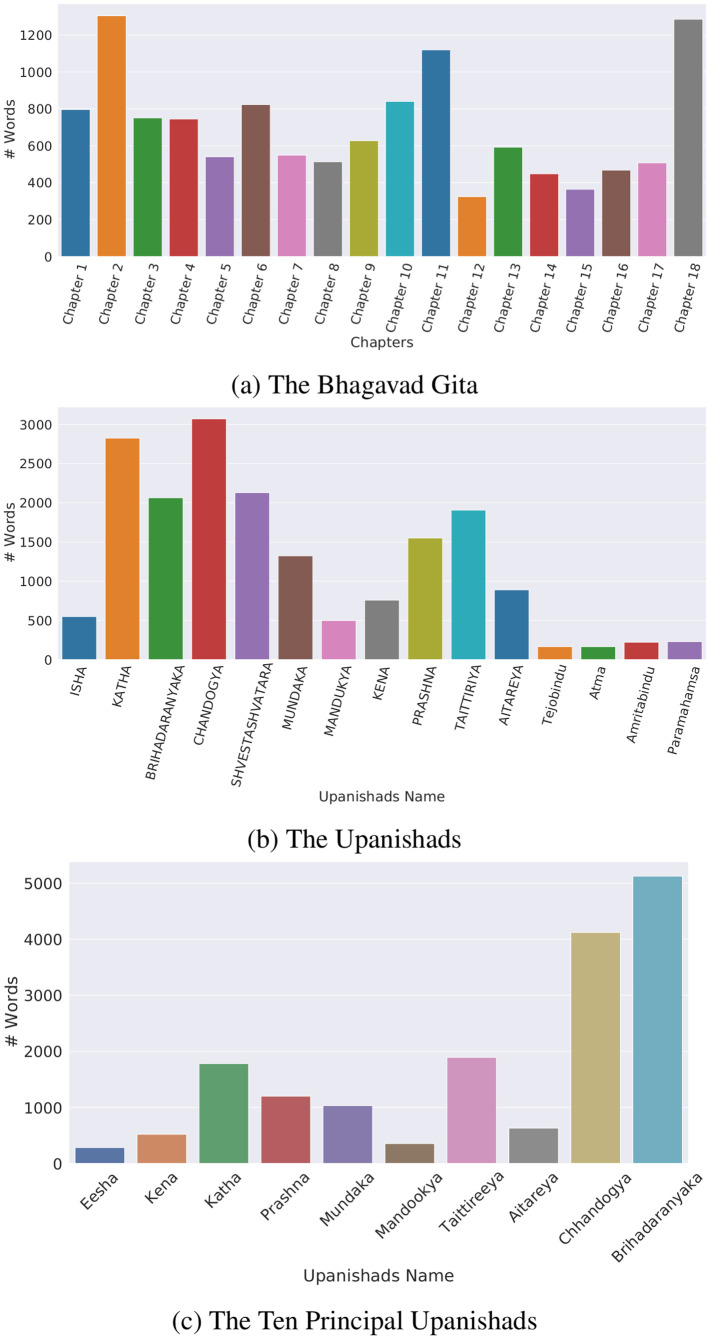
Chapter wise word count for different texts in the dataset.

Fig 2 shows the chapter-wise word count of the respective corpus. The Bhagavad Gita consists of 18 chapters, where Chapter 2 has the highest number of words, followed by Chapter 18 and Chapter 11. This is because these chapters contain relatively more verses and explain much deeper topics of Hindu philosophy. Chapter 18 contains the highest number (78 verses), followed by Chapter 2 (72 verses) and Chapter 11 (55 verses). Chapter 2 of the Bhagavad Gita discusses the Samkhya and Yoga School of Hindu Philosophy [111, 130, 131]. It teaches about cosmic wisdom (Brahm Gyan) and the methods of its attainment along with the notion of qualia (Atman/self), duty, action (karma), selfless action (karma yoga), rebirth, afterlife, and the qualities of self-realized individuals (muni) [131]. Eknath Easwaran [111] claimed this chapter as an overview of the remaining sixteen chapters of the Bhagavad Gita. Chapter 11 is also called the “Vishwa Roopa Darshana Yoga” [130] which has been translated as “The Cosmic Vision” by Eknath Easwaran [111], and “The Yoga of the Vision of the Universal” Form [130] by Swami Chinmayananda. This chapter presents the universal form (Viraat Roopa) of Lord Krishna which gave Arjuna the experience of Samadhi (enlightenment) along with the feeling of being terrified at the same time [111, 131]. When terrified, Arjuna asks about the identity of the cosmic vision of God. The reply of Lord Krishna (verse 32 of Chapter 11) came into Robert Oppenheimer’s mind when he saw the atomic bomb explode over Trinity in the summer of 1945 [111, 132]. He mentioned, “A *few people laughed, a few people cried. Most people were silent. I remembered the line from the Hindu scripture, the Bhagavad Gita; Vishnu is trying to persuade the Prince that he should do his duty and, to impress him, takes on his multi-armed form and says, Now I am become Death, the destroyer of worlds*.”

**Fig 3:**
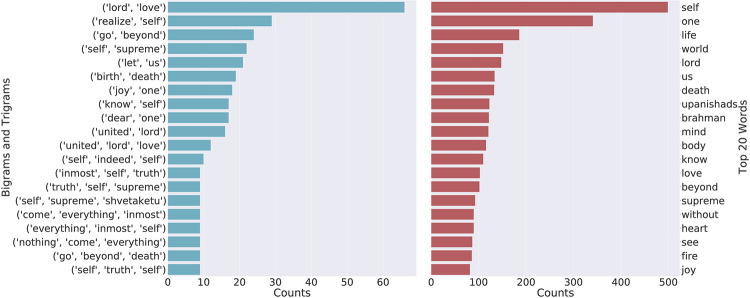
Leading bigrams and trigrams for the Bhagavad Gita and Upanishads by Eknath Easwaran.

**Fig 4:**
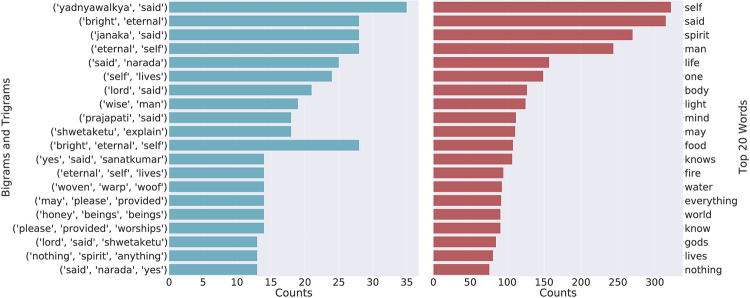
Leading bigrams and trigrams for the Ten Principal Upanishads.

The n-gram [133] is typically used to provide basic statistics of a text using a continuous sequence of words or other elements. Bi-grams and tri-grams are typical examples of n-grams. Fig 3 shows the count of the top 10 bigrams and trigrams along with the top 20 words for the Upanishads. In the case of the Upanishads, (lord, love) is the most frequent bigram which has occurred more than 60 times followed by (realize, self) and (go, beyond). In the same corpus, when we look at the trigram’s bar plot we find that (united, lord, love), (self, indeed, self) and (inmost, self, truth) are the top 3 trigrams of the corpus. Similarly, Fig 4 shows the unigrams, bigrams and trigrams of the Ten Principal Upanishads.

**Fig 5:**
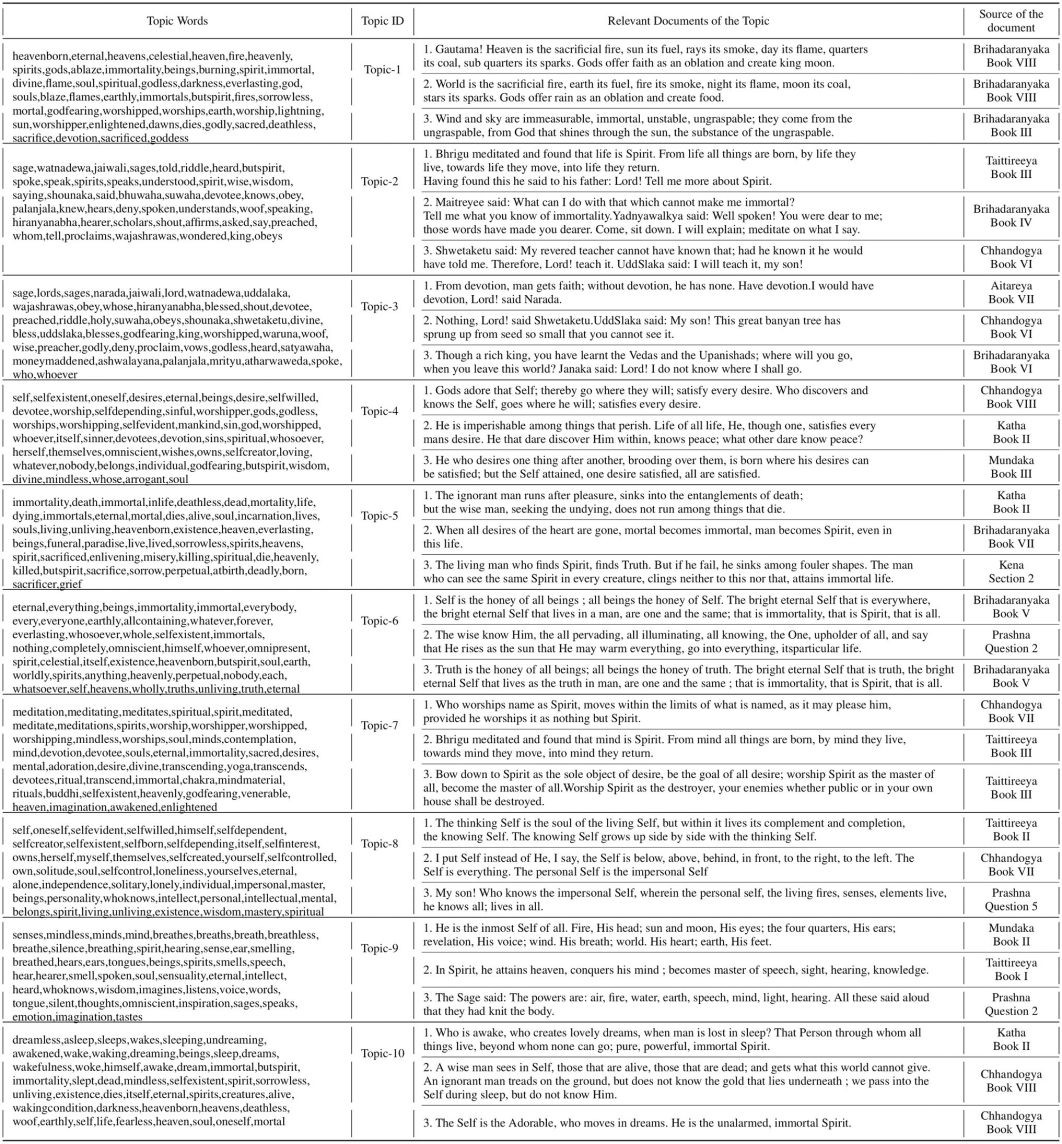
Topics of the Ten Principal Upanishads and some of their relevant documents(Model: USE-HDBSCAN-UMAP).

Although the n-grams just state the frequency of occurrence of the continuous sequence of words, they give a rough idea about the themes and topics discussed in the corpus. This can be seen in Fig 5 that a lot of topics do contain these words. We can see that ‘self’ is one of the predominant words in topic 4 and topic 8 of the Ten Principal Upanishads. We find that the entire topic is related to the theme of “Self” which is known as the Atman. In consciousness research, the Atman is referred to as the hard problem of consciousness [134–136]. Similarly, we find the words “lord”, “God” and “sage” to be predominant words in topic 1 and topic 3 of the Ten Principal Upanishads.

**Fig 6:**
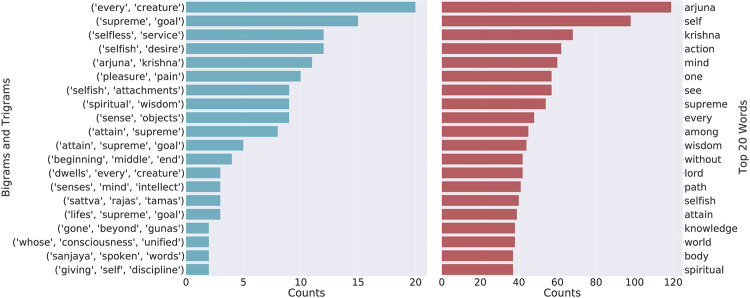
Visualisation of top 20 words, and top 10 bigrams and trigrams for the Bhagavad Gita.

**Fig 7:**
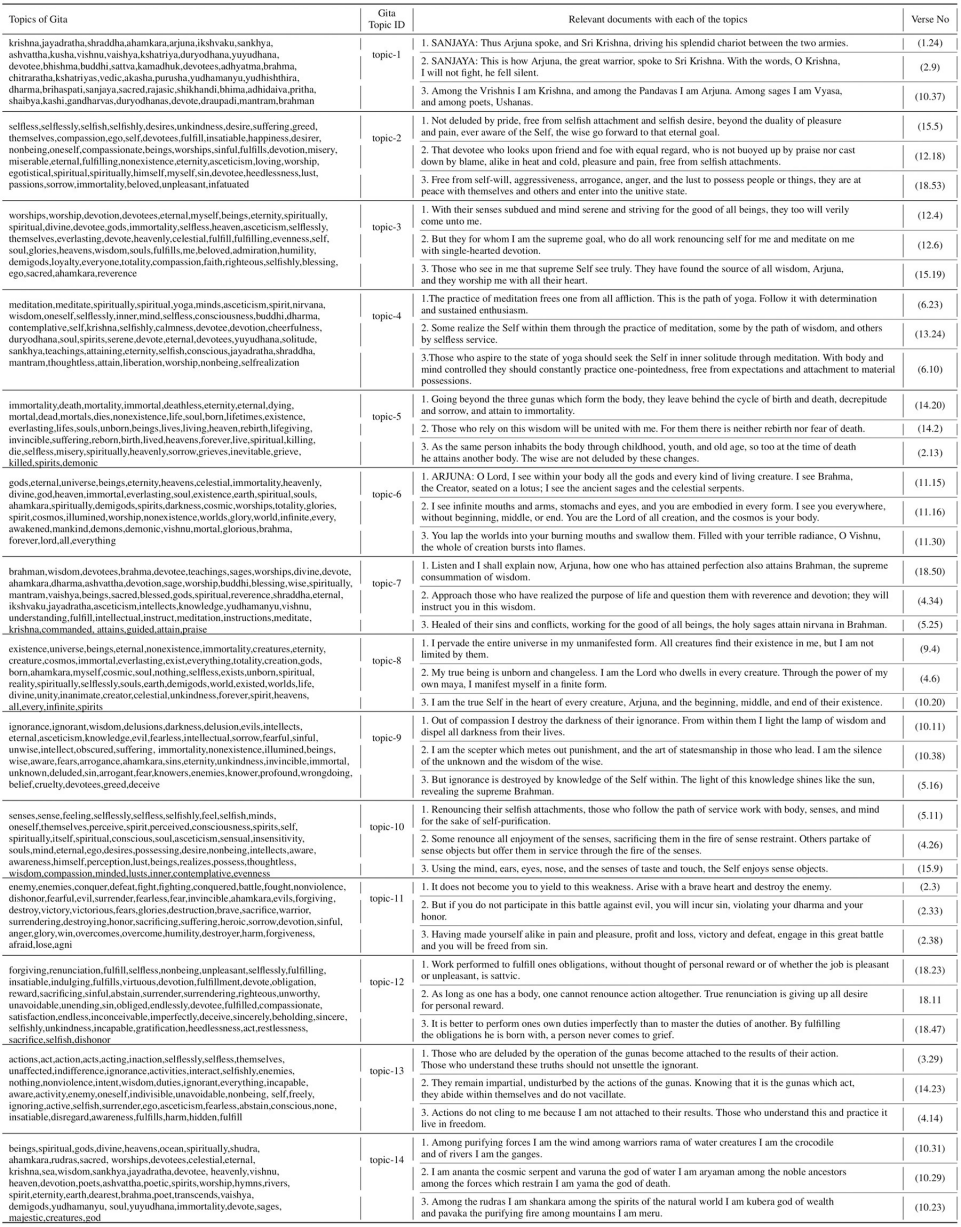
Topics of Bhagavad Gita and the most relevant documents(Model: USE-HDBSCAN-UMAP).

Fig 6 shows the bigrams, trigrams and word count for the Bhagavad Gita. We find that “arjuna, self, krishna, action” and “mind” are top 5 words of the Bhagavad Gita. Among the bigrams and trigrams, we find that (every, creature), (supreme, goal) and (selfless, service) are the top 3 bigrams while (attain, supreme, goal), (beginning, middle, end) and (dwells, every, creature) are the top 3 trigrams. Since Arjuna and Krishna are the protagonists, it is obvious for them to be among the top words of the text. We see that other than these, “self, action,” and “mind” are the prominent words that give us a basic idea about the themes that can be verified from the topics presented in Fig 7. Topic 1 of the Bhagavad Gita in Fig 7 shows all the names of the Hindu spiritual entities (deities) and we find that Krishna and Arjuna are among them. This topic also includes other entities and deities such as Jayadratha, Vishnu and Bhishma that have been mentioned by the Lord Krishna in the text. The words related to the “Self” can be seen in Topic 2 of Fig 7; hence, we can conclude that themes related to the Self are present in Topic 2 identified by our framework. We also find that Topic 13 of the Bhagavad Gita contains the words related to “action” (karma) which is also one of the top 5 words of the texts.

In terms of the individual word frequency, we find that “Self” is one of the most occurred word in all the three corpus which is a major theme of Hindu Philosophy. The Self is the translation from the Sanskrit word “Atman”, which refers to the spirit, and more precisely “qualia” as known in the definition pertaining to the *hard problem of consciousness* [137]. The Atman is also often translated as consciousness and there are schools of thought (Advaita Vedanta [138]) that sees the Atman as Brahman (often translated as God, supreme consciousness, and ultimate reality) [6, 139]. Often, it is wrongly translated to the term soul which is an Abrahamic religious concept, where humans only have the soul which excludes animals [140]. Atman on the other hand, is the core entity of all life forms and also of non-life forms in Hindu philosophy. Not only in Upanishads but it has been explained in the Bhagavad Gita as well with details. Finally, “attain supreme goal” is the most occurred trigram of the Bhagavad Gita which suggests that the Bhagavad Gita talks about attaining supreme goal with a great details along with the other philosophical topics. The Bhagavad Gita is also known as the *Karma Upanishad* or the text that focuses on the philosophy of karma (action/work) [10]. The major focus of the Bhagavad Gita is karma philosophy given a conflicting situation and the path to self realisation as the goal of life; hence, it has also been recognised as a book of leadership and management [141, 142], and psychology [143].

### 4.2 Modelling and predictions

#### 4.2.1 Topic coherence

Quantitative evaluation of topic models is one of the major challenges in natural language processing. Initially, topic models were evaluated with held-out-perplexity but it does not necessarily correlate with human evaluation [144]. A topic can be said to be coherent if all or most of the words of the topic support each other or are related [145]. The human evaluation of topic coherence is done in two ways: 1.) rating, where human evaluators rate the topic quality on a three-point topic quality score, and 2.) intrusion, where each topic is represented by its top words along with an intruding word which has a very low probability of belonging to the topic since it does not belong in the topics uncovered. It is a behavioural way to judge topic coherence and is measured by how well a human evaluator can detect the intruding word [144, 146]. Automated topic coherence metric based on *normalized pointwise mutual information*(NPMI) correlates really well with the human evaluation and interpretation of the topic coherence [146–149]. Röder et al. [150] provided a detailed study on the coherence measure and its correlation with the human topic evaluation data. We use the *topic coherence* NPMI measure (TC-NPMI) [150] as a metric to fine-tune and evaluate different models on different corpus. Eq 4 gives the NPMI for a pair of words (*w*_*i*_, *w*_*j*_) from the top N (set to 50) words of a given topic:

$$\begin{array}{c} \text{NPMI}(w_{i},w_{j})=(\frac{\text{log}\frac{P(w_{i},w_{j})+\epsilon }{P(w_{i})\cdot P(w_{j})}}{-\text{log}(P(w_{i},w_{j})+\epsilon )}) \end{array}$$ (4)

where, the joint probability *P*(*w*_*i*_, *w*_*j*_), i.e the probability of the single word *P*(*w*_*i*_) is calculated by the Boolean sliding window approach (window length of *s* set to the default value of 110). We create a virtual document and count the occurrence of the word (*w*_*i*_) or the word pairs (*w*_*i*_, *w*_*j*_), and then it is divided by the total number of virtual documents.

We use TC-NPMI as the topic-coherence measure to evaluate different topic models and tune different hyper-parameters of different algorithms. Table 4 shows the value of metric for different model on different datasets. We trained the LDA model for 200 iterations with other hyper-parameters set to the default value as given in the *gensim* [151] library. We fine-tuned the number of topic parameters to get the optimal value of TC-NPMI.

**Table 4:** Value of topic coherence metric (TC-NPMI) for different corpus.

Model	Datasets							
	Bhagavad Gita		The Upanishads		Ten Principal Upanishads		108 Upanishads	
	# Topics	TC-NPMI	# Topics	TC-NPMI	# Topics	TC-NPMI	# Topics	TC-NPMI
SBERT-UMAP-HDBSCAN	14	0.70	18	0.67	28	0.70	115	0.63
SBERT-UMAP-KMeans	14	0.72	18	0.69	28	0.73	115	0.66
USE-UMAP-HDBSCAN	14	0.73	18	0.67	32	0.73	125	0.64
USE-UMAP-KMeans	14	0.73	18	0.69	32	0.71	125	0.67
LDA	20	0.32	20	0.29	24	0.31	140	0.39

Next, we evaluate different components in the BERT-based topic model framework presented earlier (Fig 1. We develop five major approaches from our topic modelling framework which includes: 1.) SBERT-UMAP-HDBSCAN, 2.) SBERT-UMAP-KMeans, 3.) USE-UMAP-HDBSCAN, 4.) USE-UMAP-KMeans, and 5.) LDA. In Table 4, we observe that in the case of the Bhagavad Gita, the combination of USE-UMAP-KMeans gives the best TC-NPMI score on both the datasets with a very slight difference when compared to USE-UMAP-HDBSCAN and SBERT-UMAP-KMeans. Note that high TC-NPMI results indicate better results. In the case of the Upanishads, we find a similar trend. We also observe that LDA does not perform well, even after fine-tuning the number of topic parameters to optimize the topic coherence.

Although the use of KMeans for the clustering component gives the best result, we choose USE-UMAP-HDBSCAN to find the topic similarity between the Upanishads and The Bhagavad Gita in the next section. This is because HDBSCAN does not require us to specify the number of clusters, that corresponds to the number of topics, beforehand. USE-UMAP-HDBSCAN gives 18 topics for the Upanishads for the optimal value of the topic coherence mentioned in Table 4. Similarly, we get 14 topics from the Bhagavad Gita [152]. In the case of the 108 Upanishads which contains a larger number of documents when compared to the rest of the texts, we get more topics for the optimal value of topic coherence. However, we reduced the number of topics using hierarchical topic reduction [54] in some cases for example, while comparing the topic similarity of the Bhagavad Gita and the Upanishads. Since the number of documents and words are different for the different corpus as seen from Table 3, the number of topics obtained are different for different corpus. For example, in the Ten Principal Upanishads—there are 1267 documents and we got 28 topics for them at the optimal value of topic coherence. Similarly for 108 Upanishads, there are 6191 documents which give 115 topics (Table 4) for the model SBERT-UMAP-HDBSCAN at the optimal value of topic coherence.

**Fig 8:**
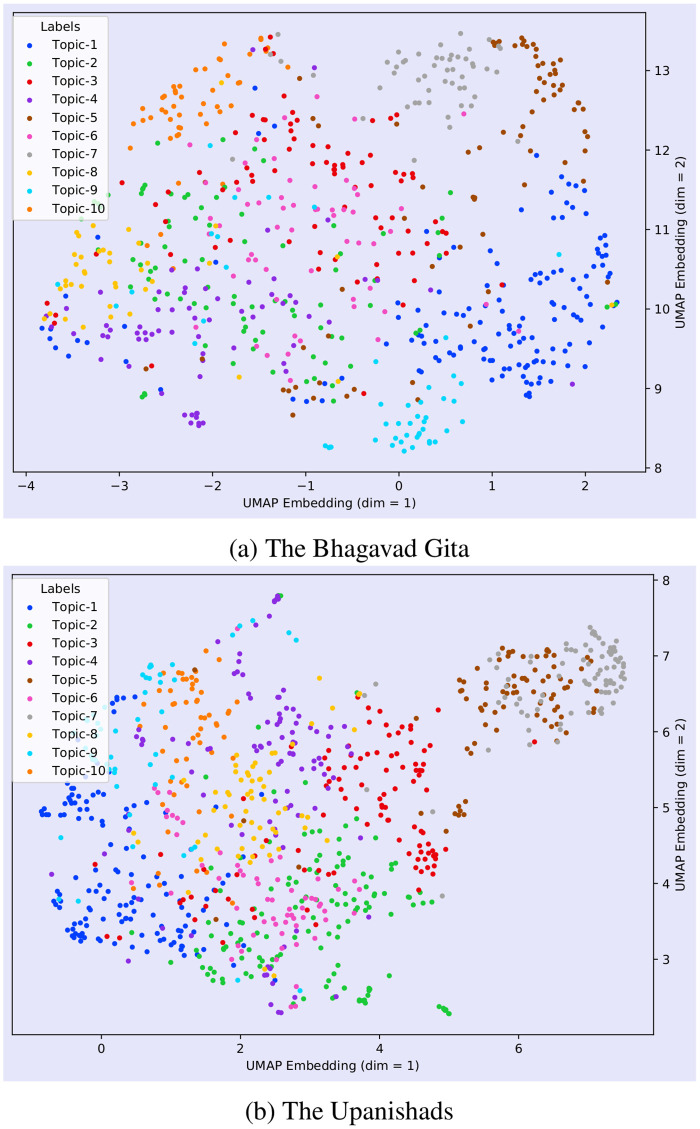
Visualization of the semantic space of the Bhagavad Gita (Eknath Easwaran) and the Upanishads (Eknath Easwaran) with topic labels.

**Fig 9:**
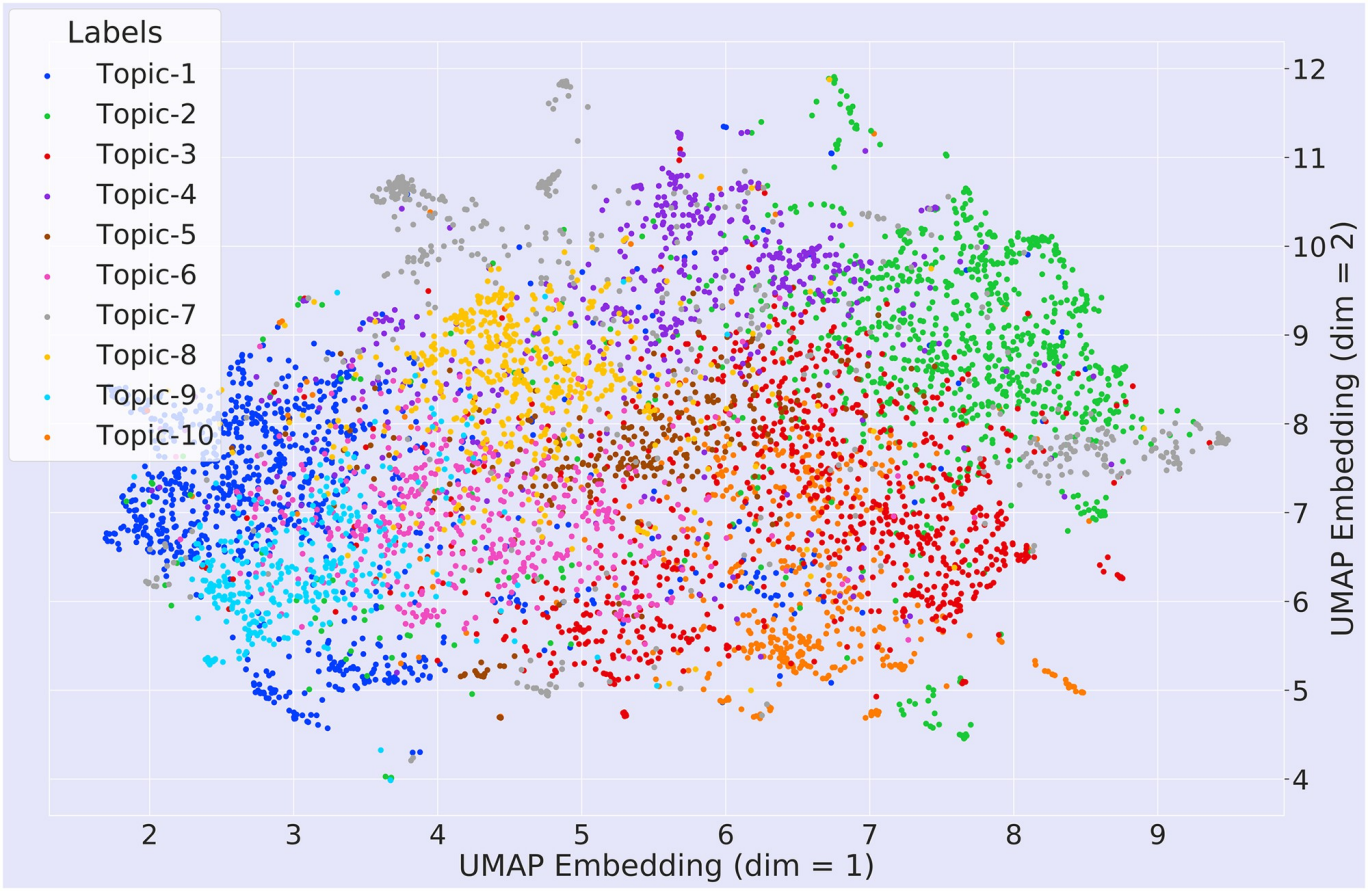
Visualisation of different topics of 108 Upanishads.

**Fig 10:**
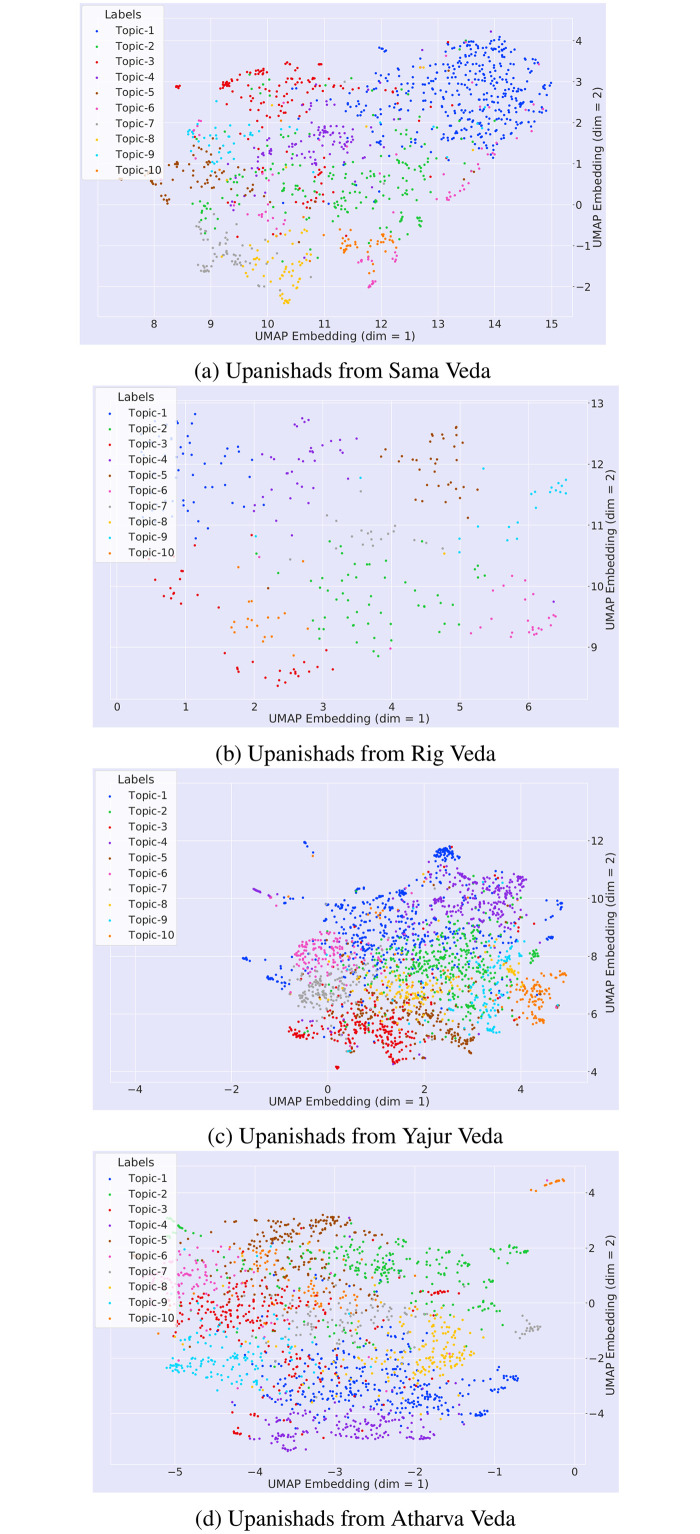
Visualization of the semantic space of different parts (based on 4 Vedas) of 108 Upanishads.

We reduced the number of topics to 10 in order to visualize the topic’s semantic space clearly while plotting the semantic space for the different topics obtained by our framework (Figs 8–10).

#### 4.2.2 Topic similarity between the Bhagavad Gita and the Upanishads

There are studies that suggest that the Bhagavad Gita summarizes the key themes of the Upanishads and various other Hindu texts [153–155]. The Bhagavad Gita along with the Upanishads and the Brahma Sutras is known as the *Prasthanatrayi* [156–160], literally meaning *the three points of departure [156], or the three sources [158]*), which makes the three foundational texts of the Vedanta school of Hindu philosophy [15, 16, 154, 155, 161]. Sargeant et al. [153] stated that the Bhagavad Gita is the summation of the Vedanta. Nicholson et al. [155] and Singh et al. [154] regarded the Bhagavad Gita as the key text of the Vedanta philosophy.

Another source which discusses a direct relationship between the Bhagavad Gita and the Upanishads is the Gita Dhayanam (also sometimes called Gita Dhyana and Dhyana Slokas) which refers to the invocation of the Bhagavad Gita) [152, 162, 163]. We note that Gita Dhayanam is an accompanying text with 9 verses used for prayer and meditation that complements the Bhagavad Gita. These 9 verses are attributed traditionally to Sri Madhusudana Sarasvati and are generally chanted by the students of Gita before they start their daily studies [162]. These verses offer salutations to various Hindu entities such as the Vyasa, Lord Krishna, Lord Varuna, Lord Indra, Lord Rudra and the Lord of the Maruta and also characterise the relationship between the Bhagavad Gita and the Upanishads. The 4th verse of the Gita Dhyanam states a direct cow and milk relationship between the Upanishads and the Gita. Eknath Easwaran [152] translated the 4th verse as “*The Upanishads are the cows milked by Gopala, the son of Nanda, and Arjuna is the calf. Wise and pure men drink the milk, the supreme, immortal nectar of the Gita*”. Although these relationships have been studied and retold for centuries, there are no existing studies that establish a quantitative measure of this relationship using modern language models.

**Fig 11:**
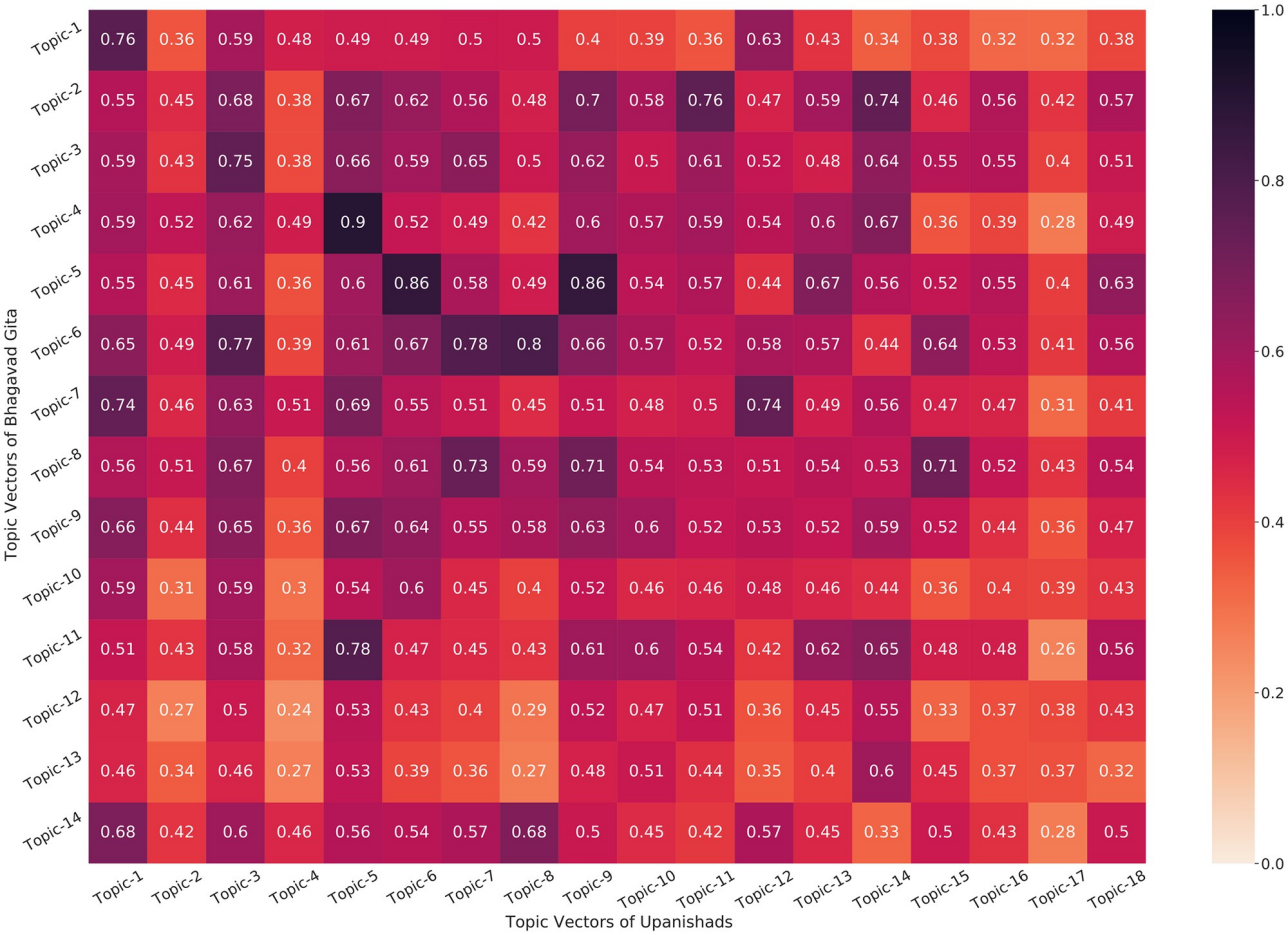
Heatmap showing the similarity between different topics of Bhagavad Gita (Eknath Easwaran) and Upanishads (Eknath Easwaran) generated from a selected approach (SBERT-UMAP-HDBSCAN).

Next, we evaluate and discuss similar relationships both quantitatively using a mathematical formulation and also qualitatively by looking at the topics generated by our models as shown in Tables 5 and 6, and Figs 5 and 7. In order to evaluate the relationship between the Bhagavad Gita and the Upanishads, we use the topics obtained to find a similarity matrix as shown in the heatmap. Note that we have two different texts (translations) of the Upanishads, i.e. by Eknath Easwaran and Sri Purohit Swami & W.B. Yeats (Ten Principal Upanisads). In Fig 11, the vertical axis of the heatmap shows the topics of the Bhagavad Gita while the horizontal axis of the heatmap represent the topics of the Upanishads. The heatmap represents the cosine similarity of the topic-vector obtained by the topic model. Therefore, in each of the topics obtained from the Bhagavad Gita, we calculate its similarity with all the topics of the Upanishads and then find the topic with maximum similarity. This operation is mathematically represented by the Eq 5a. We represent the number of topics in Gita by *N*_*gita*_ and the number of topics in Upanishads by *N*_*upan*_. In each topic $T_{i}^{gita}$ from the Bhagavad Gita, we explore and find the most similar topic from Upanishads $T_{i}^{upan}$. The topics and their similarity score can be found in Tables 5 and 6.

**Table 5:** Topics of the Bhagavad Gita(Eknath Easwaran) with most similar topics from the Upanishads(Eknath Easwaran).

Topics of Gita	Gita Topic ID	Most Similar topics in Upanishads	Upanishads Topic ID	Similarity Score
krishna,jayadratha,shraddha,ahamkara,arjuna,ikshvaku,sankhya,ashvattha,kusha,vishnu	topic-1	sage,wisdom,devotee,sages,vishnu,mahabharata,devotees,samashrava,hindu,mahavakyas,theravada	topic-1	0.76
selfless,selflessly,selfish,selfishly,desires,unkindness,desire,suffering,greed,themselves	topic-2	desires,happiness,eternal,selfless,beings,spiritual,existence,spirituality,desire,joy,eternity,buddhism	topic-11	0.76
worships,worship,devotion,devotees,eternal,myself,beings,eternity,spiritually,spiritual	topic-3	eternal,divine,deity,eternity,lords,lord,everlasting,devotional,omnipotent,gods,beings,soul,beloved	topic-3	0.75
meditation,meditate,spiritually,spiritual,yoga,minds,asceticism,spirit,nirvana,wisdom	topic-4	meditation,meditating,meditates,meditate,meditated,minds,mind,spiritually,interiorize,enlightenment,spiritual	topic-5	0.90
immortality,death,mortality,immortal,deathless,eternity,eternal,dying,mortal,dead,mortals	topic-5	immortality,death,immortal,mortality,deathless,mortal,dying,mortals,eternity,deathlessness,eternal	topic-6	0.86
gods,eternal,universe,beings,eternity,heavens,celestial,immortality,heavenly,divine,god	topic-6	celestial,sun,heavens,earth,earthly,heaven,heavenly,luminous,sunrise,sky,universe,illumined,light,illumine	topic-8	0.80
brahman,wisdom,devotees,brahma,devotee,teachings,sages,worships,divine,devote	topic-7	sage,wisdom,devotee,sages,vishnu,mahabharata,devotees,samashrava,hindu,mahavakyas,theravada,hindus,buddhi	topic-1	0.74
existence,universe,beings,eternal,nonexistence,immortality,creatures,eternity,creature,cosmos	topic-8	universe,omnipotent,eternal,cosmos,eternity,beings,cosmic,immortal,gods,celestial,deity,beyondness,god,heavens	topic-7	0.73
ignorance,ignorant,wisdom,delusions,darkness,delusion,evils,intellects,eternal,asceticism	topic-9	meditation,meditating,meditates,meditate,meditated,minds,mind,spiritually,interiorize,enlightenment,spiritual	topic-5	0.67
senses,sense,feeling,selflessly,selfless,selfishly,feel,selfish,minds,oneself,themselves,perceive	topic-10	meditation,meditating,meditates,meditate,meditated,minds,mind,spiritually,interiorize,enlightenment,spiritual	topic-5	0.78
enemy,enemies,conquer,defeat,fight,fighting,conquered,battle,fought,nonviolence,dishonor	topic-11	immortality,death,immortal,mortality,deathless,mortal,dying,mortals,eternity,deathlessness,eternal,dead,deaths	topic-6	0.60
forgiving,renunciation,fulfill,selfless,nonbeing,unpleasant,selflessly,fulfilling,insatiable,indulging	topic-12	selfs,self,selfless,oneself,himself,themselves,selfish,ego,itself,egoism,yourself,independently,ourselves,autonomic	topic-14	0.55
actions,act,action,acts,acting,inaction,selflessly,selfless,themselves,unaffected,indifference,ignorance	topic-13	selfs,self,selfless,oneself,himself,themselves,selfish,ego,itself,egoism,yourself,independently,ourselves,autonomic	topic-14	0.60
beings,spiritual,gods,divine,heavens,ocean,spiritually,shudra,ahamkara,rudras,sacred,worships	topic-14	sage,wisdom,devotee,sages,vishnu,mahabharata,devotees,samashrava,hindu,mahavakyas,theravada,hindus,buddhi	topic-1	0.68
Mean Similarity Score(AvgSim)				0.73

**Table 6:** Topics of the Bhagavad Gita(Eknath Easwaran) with most similar topics from the Ten Principal Upanishads(Shri Purohit Swami & W.B. Yeats).

Topics of Gita	Gita Topic ID	Most Similar topics in Upanishads	Upanishads Topic ID	Similarity Score
krishna,jayadratha,shraddha,ahamkara,arjuna,ikshvaku,sankhya,ashvattha,kusha,vishnu	topic-1	sage,watnadewa,jaiwali,sages,told,riddle,heard,butspirit,spoke,whoknows,thathe,speak,spirits	topic-2	0.65
selfless,selflessly,selfish,selfishly,desires,unkindness,desire,suffering,greed,themselves	topic-2	himself,self,selfexistent,oneself,desires,eternal,beings,desire,selfwilled,devotee,worship,sinful	topic-4	0.77
worships,worship,devotion,devotees,eternal,myself,beings,eternity,spiritually,spiritual	topic-3	immortality,immortal,immortals,heaven,eternal,heavenborn,heavens,heavenly,celestial,paradise	topic-14	0.70
meditation,meditate,spiritually,spiritual,yoga,minds,asceticism,spirit,nirvana,wisdom	topic-4	meditation,meditating,meditates,spiritual,spirit,meditated,meditate,meditations,spirits,butspirit	topic-7	0.80
immortality,death,mortality,immortal,deathless,eternity,eternal,dying,mortal,dead,mortals	topic-5	immortality,death,immortal,inlife,deathless,dead,mortality,life,dying,immortals,eternal,mortal,dies	topic-5	0.89
gods,eternal,universe,beings,eternity,heavens,celestial,immortality,heavenly,divine,god	topic-6	heavenborn,eternal,heavens,celestial,heaven,fire,heavenly,spirits,gods,ablaze,immortality,beings,spirit,immortal	topic-1	0.82
brahman,wisdom,devotees,brahma,devotee,teachings,sages,worships,divine,devote	topic-7	knowledge,whoknows,knowthe,wisdom,known,unknowable,knower,knowing,knew,knows,omniscient	topic-11	0.67
existence,universe,beings,eternal,nonexistence,immortality,creatures,eternity,creature	topic-8	eternal,everything,beings,immortality,immortal,everybodys,everybody,all,every,everyone,earthly,allcontaining,whatever	topic-6	0.73
ignorance,ignorant,wisdom,delusions,darkness,delusion,evils,intellects,eternal,asceticism	topic-9	knowledge,whoknows,knowthe,wisdom,known,unknowable,knower,knowing,knew,knows,omniscient	topic-11	0.71
enemy,enemies,conquer,defeat,fight,fighting,conquered,battle,fought,nonviolence,dishonor	topic-10	immortality,death,immortal,inlife,deathless,dead,mortality,life,dying,immortals,eternal,mortal,dies	topic-5	0.61
senses,sense,feeling,selflessly,selfless,selfishly,feel,selfish,minds,oneself,themselves,perceive	topic-11	self,oneself,selfevident,selfwilled,himself,selfdependent,selfcreator,selfexistent,selfborn,selfdepending,itself,selfinterest	topic-8	0.64
forgiving,renunciation,fulfill,selfless,nonbeing,unpleasant,selflessly,fulfilling,insatiable,indulging	topic-12	himself,self,selfexistent,oneself,desires,eternal,beings,desire,selfwilled,devotee,worship,sinful,worshipper,gods,godless	topic-4	0.64
actions,act,action,acts,acting,inaction,selflessly,selfless,themselves,unaffected,indifference,ignorance	topic-13	himself,self,selfexistent,oneself,desires,eternal,beings,desire,selfwilled,devotee,worship,sinful,worshipper,gods,godless	topic-4	0.56
beings,spiritual,gods,divine,heavens,ocean,spiritually,shudra,ahamkara,rudras,sacred,worships,devotees	topic-14	heavenborn,eternal,heavens,celestial,heaven,fire,heavenly,spirits,gods,ablaze,immortality,beings,burning,spirit,immortal	topic-1	0.76
Mean Similarity Score(AvgSim)				0.71

**Fig 12:**
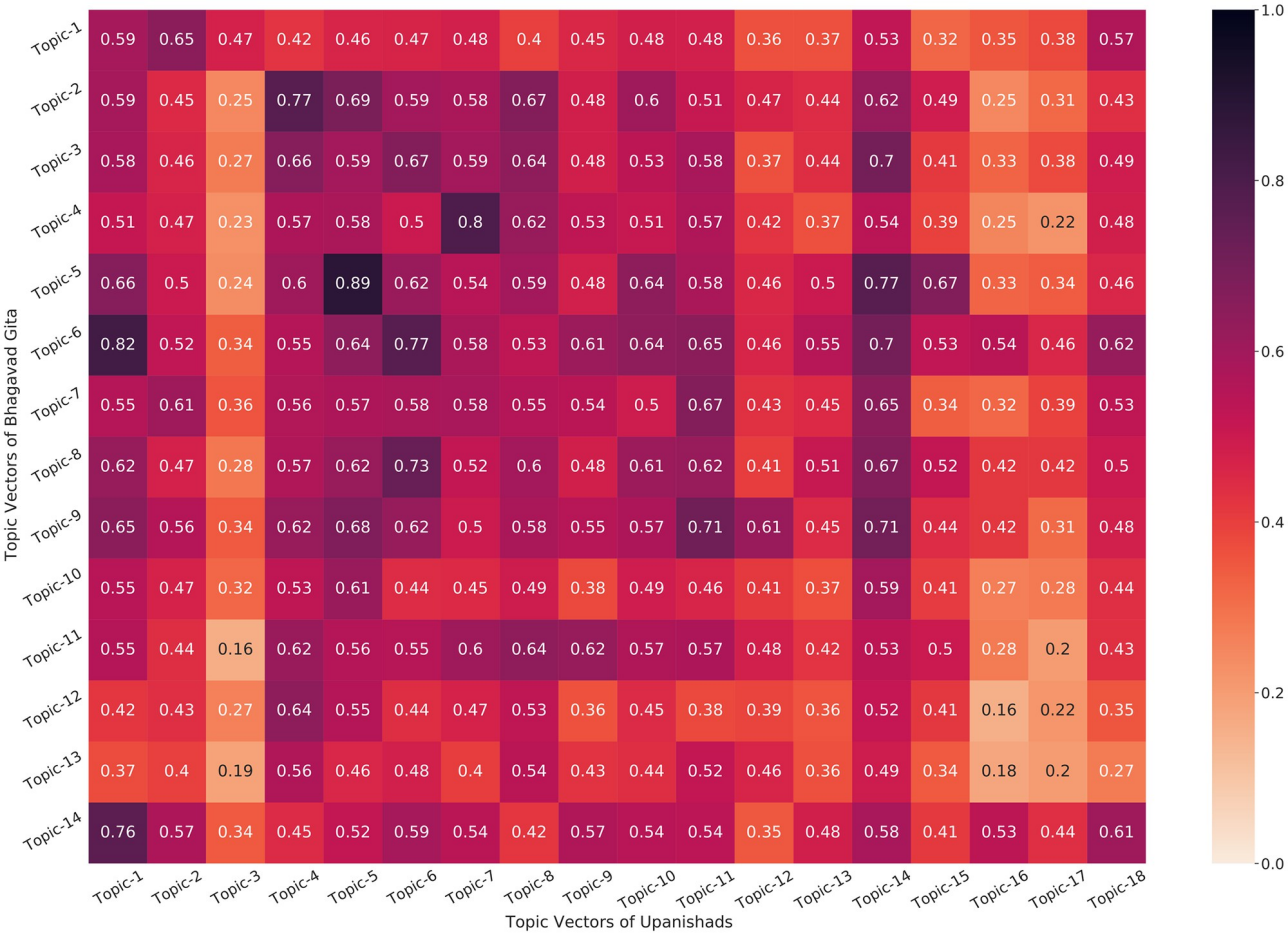
Heatmap showing the similarity between different topics of Bhagavad Gita (Eknath Easwaran) and the Ten Principal Upanishads (Shri Purohit Swami & W.B. Yeats) generated from a selected approach (SBERT-UMAP-HDBSCAN).

In Fig 11, we find that some of the highly correlated topic pairs are given as follows: (Topic-5:Gita and Topic-6:Upanishads), (Topic-4:Gita and Topic-5:Upanishads), and (Topic-5:Gita and Topic-9:Upanishads). We take an example from Table 5 to interpret (Topic-5:Gita and Topic-6:Upanishads) further. We find that Topic-5:Gita key terms refer to “immortality, death, mortality, immortal, deathless, eternity, eternal, dying, mortal, dead, mortals”. In comparison, we find Topic-6:Upanishads as “immortality, death, immortal, mortality, deathless, mortal, dying, mortals, eternity, deathlessness, eternal” and the link between them is clear. We observe a similar trend in other topic combinations with high scores, and a similar trend is also given for the other translation combination, i.e. Bhagavad Gita and the Ten Principal Upanishads given in Fig 12 and Table 6.

We observe a very high similarity in the topics of the Bhagavad Gita and two different texts of Upanishads (shown in Tables 5 and 6). These tables also show the *mean similarity score* which is given by the average of all the similarity scores as shown in Eq 5b and given below:

$$\begin{array}{c} {\text{T}}_{\text{i}}^{\text{upan}}=\overset{N_{upan}}{\underset{j=1}{\text{arg}\;\text{max}}}\;\text{Sim}(V_{i}^{gita},V_{j}^{upan}) \end{array}$$ (5a)

$$\begin{array}{c} \text{AvgSim}=\frac{\sum_{i=1}^{N_{gita}}\overset{N_{upan}}{\underset{j=1}{\text{max}}}\;\text{Sim}(V_{i}^{gita},V_{j}^{upan})}{N_{gita}} \end{array}$$ (5b)

where $V_{i}^{gita}$ and $V_{i}^{upan}$ represent the *i*^*th*^ topic vectors of the Bhagavad Gita and the Upanishads, respectively. *Sim*(.) represents the similarity measure defined by Eq 6, which is cosine similarity in our case. There are various other measures of similarity score between two vectors; however, the cosine similarity is used widely in the literature [164–166]. One of the major reasons for this is its interpretability. Note that the value of cosine similarity between any two vectors lie between 0 and 1. A value closer to 1 represents perfect similarity and a value closer to 0 represent that they are completely dissimilar.

The cosine similarity between any two vectors *U* and *V* is represented by Eq 6. Since the topic vector contains contextual and thematic information about a topic, the similarity score gives us extent of closeness of the themes and topics of the Bhagavad Gita and the Upanishads.

$$\begin{array}{c} \text{Sim}(\text{U},\;\text{V})=\text{cos}\left(\theta \right)=\frac{U\cdot V}{\left\|U\|\|V\right\|} \end{array}$$ (6)

We can observe from the Table 5 that several topics in the Bhagavad Gita are similar to the topics of the Upanishads with more than 70% similarity. We also find that topic 4 of the Bhagavad Gita is similar to topic 5 of the Upanishads (similarity of 90%). We can see that both topics contains almost similar words. Similarly, topic-5 of the Bhagavad Gita has a similarity of 86% when compared with topic 8 of the Upanishads. Both of these topics are related to immortality and death. The similarity can be observed via Table 5; for example, topic-1 of both Bhagavad Gita and the Upanishads (Eknath Easwaran) consists of the words related to Hindu deities and entities such as Krishna, Arjuna, Vishnu and Samashrava, they also have a similarity of 76%.

Fig 8 represents a visualization of the semantic space of the Bhagavad Gita and the Upanishads with given topic labels. Although we find in Table 4 that Bhagavad Gita and the Upanishads gave 14 and 18 topics respectively, we are only presenting 10 topics from both texts to have a clear visualization. Each dots in the diagram represent the two dimensional (2D) embedding of each of the documents of the corpus. These topics can be seen in Fig 7 along with some of the most relevant documents of the text with their source. Fig 7 represents the themes related to the deities and the entities of the Hindu philosophy. We can also observe that documents relevant to topic-1 have been originated form Chapters 1, 3 and 10. These all are the verses containing the name of the Hindu deities. Topic-2 of the same table encapsulate the idea of self, worship, desire and fulfilment. A similar pattern can be observed in Table 6 which represent the topics and documents of the Ten Principal Upanishads.

In Fig 9, we observe that the certain topics are separated by a large distance in UMAP embedding (dim = 1); these include Topic 1, Topic 2 and Topic 3 which imply that their themes do not have overlapping or common features. Moreover, there are some topics that have a large overlap with neighbouring topics, such as (Topic 1 and Topic 9) and (Topic 5 and Topic 3); hence, it is difficult to distinguish them implying that they have certain overlapping themes.

#### 4.2.3 108 Upanishads

Finally, we apply a selected respective topic modelling approach (USE-UMAP-HDBSCAN) from our topic modelling framework (Fig 1) for analysis of the complete 108 Upanishads. We note that the 108 Upanishads are also known as Upanishads that fall under 4 different categories identified by the four Vedas [14] (Rig Veda, Samar Veda, Yajur Veda, Artha Veda) which are known as the founding texts of Hinduism. The Rig Veda is the oldest Hindu texts written in ancient Sanskrit and believed to be remembered orally from guru-student tradition of mantra-recital [167] thousands of years before being written down [13]. It has been difficult to translated and understand significance of certain aspects of the Vedas since it has been written in ancient Sanskrit in verse form [168]. The Upanishads are known as the texts that explain the philosophy of the Vedas and also known as the concluding chapters that have been added to the four Vedas [169]. Table 7 gives information about how the 108 Upanishads have been grouped according to their historical relevance to the respective Vedas. Fig 10 presents visualization of the semantic space of different parts (divided by 4 Vedas as shown in Table 7) of 108 Upanishads.

**Table 7:** Classification of 108 Upanishads based on the four key Vedas. Note that the original Yajur Veda is divided into two parts (Krishna- Yajur-Veda and Sukla-Yajur-Veda).

Vedas	Number	Titles (names) of the Upanishad
Atharva-Veda	31	Prasna,Mundaka,Mandukya, Atahrvasiras,Atharvasikha,Brihajjabala,Devi Nrisimhatapini,Naradaparivrajaka,Sita,Sarabha,Tripadvibhuti-Mahanarayana,Ramarahasya,Ramatapini,Sandilya,Paramahamsaparivrajaka,Annapurna,Surya,Atma,Pasupatabrahmana,Parabrahma,Tripuratapini,Bhavana,Bhasmajabala,Ganapati,Mahavakya,Gopalatapini,Krishna,Hayagriva,Dattatreya,Garuda
Krishna-Yajur-Veda	32	Kathavalli,Taittiriyaka,Brahma,Kaivalya,Svetasvatara, Garbha,Varaha,Akshi Narayana,Amritabindu,Amritanada,Kalagnirudra,Kshurika,Sarvasara Sukarahasya,Tejobindu,Dhyanabindu,Brahmavidya,Yogatattva,Dakshinamurti,Skanda,Sariraka,Yogasikha,Ekakshara,Avadhuta,Katharudra,Rudrahridaya,Yoga-kundalini,Panchabrahma,Pranagnihotra,Kalisamtarana,Sarasvatirahasya
Sukla-Yajur-Veda	19	Isavasya,Brihadaranyaka,Jabala,Hamsa,Paramahamsa,Paingala,Bhiksu,Tarasara Mantrika,Niralamba,Trisikhibrahmana,Mandalabrahmana,Turiyatita,Subala Satyayani,Muktika,Advayataraka,Adhyatma,Yajnavalkya
Sama Veda	16	Kena,Chandogya,Aruni,Maitrayani,Maitreya,Vajrasuchika,Jabali Rudrakshajabala, Yogachudamani,Vasudeva,Savitri,Darsana Mahat,Sannyasa,Avyakta,Kundika
Rig Veda	10	Aitareya,Kaushitakibrahmana,Nadabindu,Atmabodha,Nirvana,Mudgala,Akshamalika,Tripura,Saubhagyalakshmi, Bahvricha

## 5 Discussion

The high level of semantic and topic similarity between the Bhagavad Gita and the different sets of the Upanishads by the respective authors is not surprising. It verifies well known thematic similarities as pointed out by Hindu scholars such as Swami Vivekananda [170] and western scholars [16]. The Bhagavad Gita is well known as the central text of Hinduism that summarizes the rest of the Vedic corpus. The Bhagavad Gita is a conversation between Lord Krishna and Arjuna in a situation where Arjuna has to go to war. The Bhagavad Gita is a chapter from the Mahabharata that uses a conflicting event to summarize philosophy of the Upanishads and the Vedic corpus. The Mahabharata is one of the oldest and longest texts written in verse form in Sanskrit which describes a historical event (118,087 sentences, 2,858,609 words) [171]. We note that most of the Hindu ancient and scared texts have been written in verse form so that they can be sung and remembered through an oral tradition given an absence of a writing system.

The goal of Lord Krishna was to motivate Arjuna to do his duty (karma) and go to war to protect ethical standards (dharma) in the society. Krishna, in the Bhagavad Gita begins by renouncing his duties as a warrior. We note that the Mahabharata war is known to have taken place after the Vedas were composed. Note that by composition, it does not mean that these texts were written, they became key mantras that were remembered through a guru-student tradition for thousands of years. There are accounts where the Vedas have been mentioned in the Mahabharata. Hence, Krishna is known as a student of the Vedic corpus which also refers to the entire library of Hindu science, literature, history and philosophy. Therefore, the topics in the Upanishads were well known by Lord Krishna and he may have merely used some of the themes to highlight about themes of duty, ethics (dharma) and work (karma) in order to motivate Arjuna to do his duty at the time of need; otherwise, his side (Pandavas) would lose the war to the opposition (Kauravas). The Mahabharata war has blood relatives on opposing sides of the war battleground known as Kurushetra; hence, it was difficult for Arjuna to make a decision—either to fight for dharma or become a yogi (mystic).

Table 5 further compares the topics of the Bhagavad Gita with the Upanishads. We can observe that each of the topic encapsulate some of the ideas expressed in selected verses shown in Figs 5 and 7. If a topic of the Gita and the Upanishads have very high similarity, this represents the fact that the ideas encapsulated by the topics of the Gita and the Upanishads are almost the same. In Table 5, we can observe that topic 4 of the Bhagavad Gita and topic-5 of the Upanishads have a similarity of 90%, this can be seen from the topics also they are representing the similar themes that are related to the ideas of meditation, yoga and spirituality. Similarly, we observe that topic-5 of Gita have a similarity score of 86%, when compared with topic-6 of the Upanishads. Here, we can also observe that both topics encapsulate similar ideas of death, mortality and immortality. Similar ideas can be observed in Table 6 as well, where the topics of the Bhagavad Gita are compared with the topics of the Upanishads.

**Fig 13:**
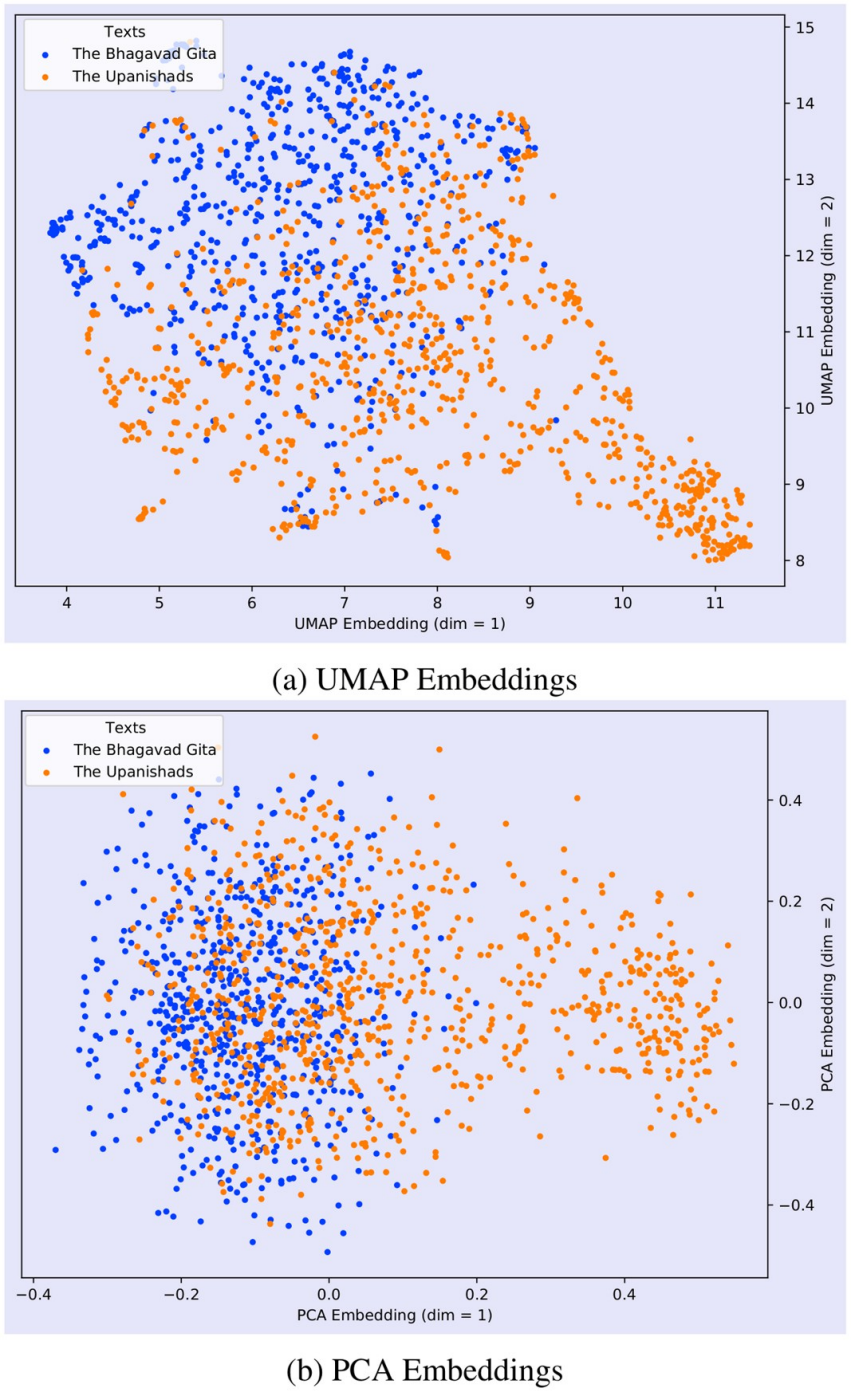
Comparison of dimensionality reduction and visualisation by PCA and UMAP for the combined semantic space of the Bhagavad Gita (Eknath Easwaran) and the Upanishads (Eknath Easwaran). Note that the PCA has 4.4% explained variance ratio for dim = 1 and 3.7% for dim = 2, taken from total of 500 dimensions in original data.

Fig 8 depicts a representation of the semantic space of the Bhagavad Gita and the Upanishads with topic labels. It represents the lower dimensional embedding of the very high dimensional document vectors. In Fig 8, we represented only 10 topics in order to retain the clarity of the diagram. Fig 13 shows the UMAP and PCA embedding of the entire document. In order to generate this plot, we first created the embeddings of each documents and then reduced the embedding to 2D by using PCA and UMAP. After reducing the dimension, we assigned the labels (*Gita*, and the *Upanishads*) based on the corpus. Fig 13 shows that low-dimensional embeddings reveals very clear overlaps across the documents.

Even with the presence of translation bias by considering two different translations of the Upanishads, our results demonstrate a very high resemblance between the topics of these two texts, with a mean cosine similarity of more than 70% between the topics of the Bhagavad Gita and those of the Ten Principal Upanishads. Eight of the fourteen topics extracted from the Bhagavad Gita have a cosine similarity of more than 70% with the topics in the Ten Principal Upanishads, which can also be seen in Table 6, where 3 topics have a similarity of more than 80%. When considering the translation of both texts by same author as in the case of the Bhagavad Gita [111] and the Upanishads [112], we see that average similarity increase to 73% with 9 out of 14 topics having more than 70% similarity and 3 of them having a similarity of more than 80%. We also found that the topics generated by the BERT-based models show very high coherence when compared to LDA. Our best performing model gives a coherence score of 73% on the Bhagavad Gita [111], 69% on the Upanishads [112], 73% on the Ten Principal Upanishads [113] and 66% on the 108 Upanishads.

Further extension can be done by taking the other translations into consideration. *The Ten Principal Upanishads* [113] published in 1938, was translated by the Irish poet William Butler Yeats and Hindu guru Shri Purohit Swami. The translation process occurred between the two authors throughout the 1930s, and this book has been claimed as one of the final works of William Butler Yeats [172]. We note that Shri Purohit Swami has also translated the Bhagavad Gita; hence, this would be a good companion with Eknath Eashwaren for the respective texts. These extensions could help in refining the proposed framework.

Moreover, in terms of the mythological texts and epics, there are various texts such as the Vishnu Purana, Shiv Purana out of the 18 different Puranas that have underlying topics that are similar. In this study, we focused on philosophical texts, while in future studies, there can be scope for topic modelling from selected texts in the Puranas. The framework can also be used to study texts from other religions, along with n non-religious and non-philosophical texts. Furthermore, it can be used to study themes expressed in modern poems and songs and also be used to compare different religions and time frames, i.e how the themes changes over different centuries, during a war or a pandemic (such as the COVID-19).

We note that as a perspective, there exists specialised BERT pre-trained models such as those for medicine and law [173–178], but there is nothing yet developed for philosophy. Hindu philosophy is distinct and has terms and ideas that are not present in other philosophical areas (such as western philosophy). Hence, we need specialised pre-trained BERT model for Hindu philosophy which can provide better predictions in related language tasks since it will have better knowledge-base. This work can further be improved using language models for the native Sanskrit text. We intend to explore topic models after building BERT-based language models for Hindu philosophy and literature written in Sanskrit.

Further extension can be done by taking the other translations into consideration. *The Ten Principal Upanishads* [113] published in 1938, was translated by the Irish poet William Butler Yeats and Hindu guru Shri Purohit Swami. The translation process occurred between the two authors throughout the 1930s, and this book has been claimed as one of the final works of William Butler Yeats [172]. We note that Shri Purohit Swami has also translated the Bhagavad Gita; hence, this would be a good companion with Eknath Eashwaren for the respective texts. These extensions could help in refining the proposed framework. We note that our previous work focused on semantic and sentiment analysis of the Bhagavad Gita translations [69]. Augmenting semantic and sentiment analysis to our proposed topic modelling framework can provide more insights to the meaning behind the philosophical verses. We plan to build our models in a similar fashion and investigate their variations for texts in three different languages: Hindi, English, and Sanskrit. Finally, post verification study is needed where Sanskrit expert and Hindu philosophers can study the topics uncovered by the proposed framework.

The Bhagavad Gita and the Upanishads are considerably large texts in the content of religious and philosophical texts. However, the proposed framework can be used for larger corpus such as modelling overlapping topics around the Mahabharata and the Puranas, which are texts that are magnitudes larger than the ones considered in this study. However, we note that the Bhagavad Gita and Upanishads, although smaller in size are known as texts that are philosophical while the Mahabharata is an epic narrative poem describing actual events in history. In future work, there can be a detailed study of the topics uncovered with a discussion of related texts in Vedic studies that relate to morphology, lexicography, grammar (patterns in sentences), meter (lengthy sentences), and phonology (sound system), etc. Furthermore, we need to create processed benchmark text datasets for Indian languages that can benefit NLP applications associated with Indian languages.

## 6 Conclusions

We presented a topic modeling framework for Hindu philosophy using state-of-art deep-learning based models. The use of such technique for studying Hindu texts is relatively novel; however, computational and statistical approaches have been used in the past. The major goal of the study was to link the topics from the Upanishads with the Bhagavad Gita. The representation of the low-dimensional embeddings presented in this work reveals an overlap between the Upanishads and the Bhagavad Gita’s topics. Given the importance of religious literature to a community, employing computational models to verify any of its old and traditional philosophical principles demonstrates the scientific nature of the literature and religion.

The major limitation of our study is due to the translation bias, which is not present when we use texts from the same translator. Hence, we selected the Upanishads and Bhagavad Gita by Eknath Easwaren in order to limit the bias. However, if we consider the complete 108 Upanishads which is translated by various authors, the translation bias remains. Moreover, the style and language of the translations not only depend on the translator, but also on the era of the translation. In the case of the 108 Upanishads, a group of translators have contributed which creates further biases. However, in terms of topics uncovered, we find a consistent set of topics that well alight with the respective texts, after manually verifying it.

Despite the fact that the idea of the Gita being the essential extract of the Upanishads has been written and researched in Hindu philosophical literature for generations, no attempt has ever been made to substantiate this facts using computational and scientific methodologies. Our research presents a novel way for applying modern deep learning-based methods to a centuries-old philosophical narratives.
